# Genomic and Metabolomic Analyses of the Medicinal Fungus *Inonotus hispidus* for Its Metabolite’s Biosynthesis and Medicinal Application

**DOI:** 10.3390/jof8121245

**Published:** 2022-11-25

**Authors:** Rui-qi Zhang, Xi-long Feng, Zhen-xin Wang, Tian-chen Xie, Yingce Duan, Chengwei Liu, Jin-ming Gao, Jianzhao Qi

**Affiliations:** 1 Shaanxi Key Laboratory of Natural Products & Chemical Biology, College of Chemistry & Pharmacy, Northwest A&F University, Yangling, Xianyang 712100, China; 2 Key Laboratory for Enzyme and Enzyme-like Material Engineering of Heilongjiang, College of Life Science, Northeast Forestry University, Harbin 150040, China

**Keywords:** medicinal macrofungi, chromosomal-level assembly, biosynthetic potential, bioactive metabolite

## Abstract

*Inonotus hispidus* mushroom is a traditional medicinal fungus with anti-cancer, antioxidation, and immunomodulatory activities, and it is used in folk medicine as a treatment for indigestion, cancer, diabetes, and gastric illnesses. Although *I. hispidus* is recognized as a rare edible medicinal macrofungi, its genomic sequence and biosynthesis potential of secondary metabolites have not been investigated. In this study, using Illumina NovaSeq combined with the PacBio platform, we sequenced and *de novo* assembled the whole genome of NPCB_001, a wild *I. hispidus* isolate from the Aksu area of Xinjiang Province, China. Comparative genomic and phylogenomic analyses reveal interspecific differences and evolutionary traits in the genus *Inonotus*. Bioinformatics analysis identified candidate genes associated with mating type, polysaccharide synthesis, carbohydrate-active enzymes, and secondary metabolite biosynthesis. Additionally, molecular networks of metabolites exhibit differences in chemical composition and content between fruiting bodies and mycelium, as well as association clusters of related compounds. The deciphering of the genome of *I. hispidus* will deepen the understanding of the biosynthesis of bioactive components, open the path for future biosynthesis research, and promote the application of *Inonotus* in the fields of drug research and functional food manufacturing.

## 1. Introduction

Fungi that are edible and therapeutic play an essential part in human food and traditional medicine. *Inonotus hispidus* (Bull.) P Karst. (Hymenochaetaceae) is a well-known edible and medicinal mushroom with a long history as a health food and ancient folk medicine in Europe [1] and East Asian countries, especially China. *Inonotus hispidus* is an annual facultative parasitic fungus with a hairy fruiting body referred to as a shaggy bracket [2] or shaggy polypore.

*Inonotus hispidus* parasitizes mostly deciduous trees, preferring to parasitize mulberry, ash, elm, and oak [3,4], which are extensively spread in the Northeast regions and Xinjiang province of China. Furthermore, *I. hispidus* is a characteristic white rot Basidiomycete.

*Inonotus hispidus* has traditionally been used as a medicinal mushroom [5]. According to Chinese herbal books Shennong’s Classic of Materia Medica and Compendium of Materia Medica, the ancient residents of the old Yellow River valley referred to *Inonotus* mushrooms as “*Sanghuang*” [6], which were traditional medicinal mushrooms used to heal tumors. *Inonotus hispidus* is an indigenous medicine used by the local people of Xinjiang to cure stomach ulcers, indigestion, diabetes, and specific cancer, and it is frequently used to treat dyspepsia in Northeast China [3]. A large number of phytochemical and pharmacological investigations have revealed that *I. hispidus* is rich in metabolites such as polyphenols [4,5,7,8,9,10], triterpenoids [7,8,11], and polysaccharides [12], which have anti-cancer [6,13,14], antioxidant [5,7,15,16,17], antimicrobial [16,18,19,20], immunomodulatory activities [7,21,22], as well as inhibitory activities against lipase [23,24], *α*-glycosidase [25], and GST [18].

Rapidly advancing DNA sequencing technologies are making the genomic information of macrofungi more accessible. The genomes of valuable and rare edible medicinal fungi have been published and analyzed, including *Ganoderma lucidum* [26,27], *Antrodia cinnamomea* [28], *Hericium erinaceus* [29], *Inonotus obliquus* [30], and *Laetiporus sulphureus* [31], these genomic analyses are improving our understanding of their mating types, nutritional patterns, active compound mining, biosynthetic pathways, high-yield cultivation, and population genetics research, as well as furthering their medicinal value and the health industry.

The traditional Chinese medicinal fungus “*Sanghuang*” is a collective term for a group of fungi with similar pharmacological properties and morphological characteristics according to ancient medical literature. Despite the fact that fungal taxonomy specialists have developed a distinct genus of *Sanghuangporus* and described its members [32], the significant morphological similarity makes distinguishing between *I. hispidus* and *Sanghuangporus* species challenging. One effective way to overcome this dilemma is to distinguish them at the molecular level by genome sequencing. Although *I. hispidus* has significant medicinal and culinary properties, the National Center for Biotechnology Information (NCBI) database records few available nucleotide sequences on *I. hispidus*. The existing gene sequence resources are insufficient for the biological study of *I. hispidus* at the molecular level.

Herein, we provide for the first time the whole genome sequence of *I. hispidus* at the chromosomal level. On this basis, the evolutionary state of the *Inonotus* genus, as well as their genome shrinkage and expansion, were investigated using comparative genomic analysis. The genes involved in the mating system, carbohydrate metabolism, and polysaccharide synthesis were screened, and candidate genes for secondary metabolites biosynthesis were examined. Furthermore, differences in the chemical composition and content between fruiting bodies and mycelium and specific metabolites were identified with the help of molecular networks of metabolites. This work fills the gap in the genome of *I. hispidus* and advances our understanding of the genome of medicinal, edible macrofungi.

## 2. Materials and Methods

### 2.1. Fungal Strain and Strain Culture

**Figure 1:**
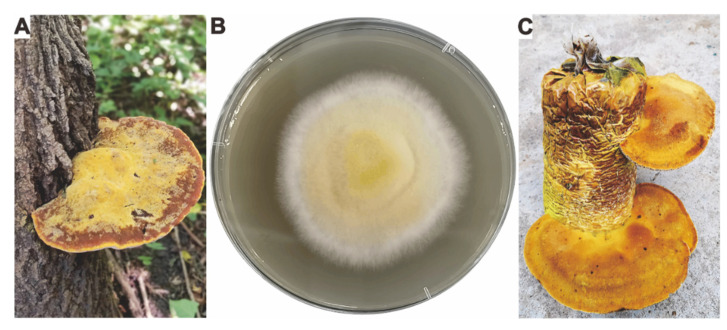
Morphologic photograph of the strain *I. hispidus* NPCB_001. (**A**) The morphologic photograph of the wild fruiting body, (**B**) mycelium growing on PDA for four days, (**C**) mature, cultivated fruiting bodies of the strain *I. hispidus* NPCB_001.

Fresh wild fruiting bodies of *I. hispidus* (Figure 1A) were used for tissue isolation, and surface sterilized fruiting bodies were cultivated on Potato Dextrose Agar (PDA) plate for 3–4 days to obtain culturable mycelium (Figure 1B). The artificially cultivated fruiting body (Figure 1C) of *I. hispidus* was obtained on a wood chips-based medium. The identified mycelium of *I. hispidus* NPCB_001 was deposited in Shaanxi Key Laboratory of Natural Products & Chemical Biology, College of Chemistry & Pharmacy, Northwest A&F University.

### 2.2. Genome Sequencing, De Novo Assembly, and Annotation

#### 2.2.1. Extraction of Genome DNA

Fresh mycelium of *I. hispidus* NPCB_001 was cultured in PDB medium (200 rpm, 25 °C) for one week to obtain an acceptable quantity of mycelia. In order to acquire fresh and clean mycelium, mycelium was collected by centrifugation, rinsed twice with sterile water, then centrifuged to remove water. The genomic DNA was isolated using the sodium dodecyl sulfate (SDS) technique after the mycelium was ground with liquid nitrogen and tested for integrity using agarose gel electrophoresis.

#### 2.2.2. *De Novo* Sequencing

Genomic DNA was end-repaired, A-tails added, sequencing junctions added, purified, and PCR amplified. High-quality bulk DNA was gathered and tested for purity, concentration, and integrity before being used to generate libraries. Quantification and quality checks were then performed using Qubit 2.0 to ensure library quality. The genome of *I. hispidus* NPCB_001 was sequenced using the PacBio Sequel long-read sequencing and Illumina NovaSeq platforms with the 20-kb and 350-bp insert sizes, respectively. The NECAT (https://github.com/xiaochuanle/NECAT) (accessed on 1 September 2022) was used to fix genome errors, and splicing was performed to provide the initial splicing result. The splicing result from third-generation sequencing data was then subjected to two rounds of error correction using Racon v1.4.7 (https://github.com/isovic/racon) (accessed on 1 September 2022), followed by two rounds of Pilon. The final assembly result was determined after mistake correction and heterozygosity elimination. The final genome assembly results and related data of *I. hispidus* NPCB_001 were submitted to NCBI under the BioProject JANBPQ000000000, BioSample SAMN29577933, and GenBank GCA_024712875.1, respectively.

#### 2.2.3. Gene Prediction and Annotation

The BRAKER v2.1.4 (https://github.com/Gaius-Augustus/BRAKER) (accessed on 1 September 2022) was primarily used to predict gene sequences. Thereafter, GeneMark-EX was used to train the model, and AUGUSTUS (https://github.com/Gaius-Augustus/Augustus) (accessed on 1 September 2022) was used to forecast ORFs. INFERNAL v1.1.2 (https://github.com/EddyRivasLab/infernal) (accessed on 1 September 2022) was used to predict and categorize ncRNA based on the Rfam database. After integrating the rebase library, RepeatModeler v1.0.4 (https://github.com/Dfam-consortium/RepeatModeler) (accessed on 1 September 2022) was used to generate its own repeat library, and RepeatMasker v4.0.5 (https://github.com/rmhubley/RepeatMasker) (accessed on 1 September 2022) was used to annotate the repetitive genomic sequence. To annotate the gene products, BLAST searches of non-redundant protein sequences from the NCBI, Swiss-Prot, COG, and KEGG databases were performed.

### 2.3. Comparative Genomics Analysis

McscanX (https://opensourcelibs.com/lib/mcscanx) (accessed on 1 September 2022) was used to analyze and visualize genome collinearity. Single-copy genes were used to undertake comparative genomic analysis within *Inonotus* species, which was visualized using jVenn (http://jvenn.toulouse.inra.fr/app/index.html) (accessed on 1 September 2022). Ks calculations were carried out on two *Inonotus* species. ParaAT 2.0 (https://github.com/wonaya/ParaAT) (accessed on 1 September 2022) was used to convert the homologous protein sequence ID lists to CDS lists. Homologous sequence pairings were estimated using KaKs Calculator 3.0 (https://ngdc.cncb.ac.cn/biocode/tools/BT000001) (accessed on 1 September 2022) and displayed using Rstudio v4.20.

### 2.4. Phylogenomic Analysis

Phylogenetic analysis was performed with the *Inonotus* strains and 45 other representative strains of Basidiomycetes. Single-copy homologous genes were identified using OrthoFinder v2.5.4 (https://github.com/davidemms/OrthoFinder) (accessed on 1 September 2022) with the parameters “-S diamond -M msa -T raxml-ng”. MCMC tree (http://abacus.gene.ucl.ac.uk/software/paml.html) (accessed on 1 September 2022) was utilized to predict divergence time with a total of 520 single-copy orthologue sequences of 24 strains. Several groups of recent ancestor divergence times were queried as calibrated points in timetree.org (http://www.timetree.org/) (accessed on 1 September 2022), (*Hericium alpestre* vs. *Stereum hirsutum* 91.8–195.5 MYA, *Marasmius oreades* vs. *Lentinula edodes* 76.9–81.2 MYA, and *Ganoderma sinense* vs. *Laetiporus sulphureus* 99–152.5 MYA).

### 2.5. CAZy Family Analysis and Structural Prediction

The database CAZy (http://bcb.unl.edu/dbCAN2/)(accessed on 7 July 2022) was used to annotate and class the genes encoding carbohydrate-active enzymes (CAZymes) from the genomes of *I. hispidus* NPCB_001 and other white-rot fungi with HMMER 3.2.1 (filter parameter e-value < 1 × 10^−5^; coverage > 0.35). A bubble plot of CAZyme analysis for *I. hispidus* was created via the Complex Heatmap package in Rstudio v4.20.

The protein structures of four CAZy members (g3766.t1, g4459.t1, g6707.t1, and g8693.t1) with bifunctional domains were predicted using SWISS-MODEL (http://swissmodelexpasy.org/) (accessed on 15 September 2022) and/or trRosetta (http://yanglab.nankai.edu.cn/trRosetta/) (accessed on 15 September 2022), and visualized by PyMol 2.4. The overly long sequence of g8693.t1 (2367 αα) prevented its second GH structural domain from being predicted.

### 2.6. Predictive Analysis of Candidate Genes for Secondary Metabolites

The biosynthetic gene clusters (BGCs) for secondary metabolite were predicted using antiSMASH 6.1(https://antismash.secondarymetabolites.org/) (accessed on 11 July 2022) with default parameters. The acquired BGCs were manually validated using PSI-BLAST (https://www.ebi.ac.uk/Tools/sss/psiblast/) (accessed on 1 September 2022) to verify the expected findings. BIG-SCAPE 1.1.0 (https://github.com/medema-group/BiG-SCAPE) (accessed on 1 September 2022) was used to create a BGC network between predicted and confirmed BGCs in the MiBIG1.4 database using cutoffs of 0.75, and the BGC network was visualized using Cytoscape3.9.1. From the anticipated terpene synthases, sesquiterpene synthases (STSs) were chosen for further investigation. The phylogenetic tree of STSs was built using 15 predicted STSs from *I. hispidus* NPCB_ 001 and identified STSs from Basidiomycota. Eight STSs on the Clade III cluster were further analyzed for identity matrix using the online web-tool https://www.ebi.ac.uk/Tools/msa/clustalo/ (accessed on 1 September 2022).

Five nonribosomal peptide synthase-like enzymes (NRPS-likes) from the strain NPCB_001 were used to construct a phylogenetic tree using the maximum likelihood method for clustering analysis with the identified PKSs from fungi in UniProt (https://www.uniprot.org) (accessed on 30 September 2022). The same method was used to analyze two polyketide synthases (PKSs). The PKS used for the clustering analysis was identified as PKS from Basidiomycota in UniProt (accessed on 12 July 2022).

### 2.7. Prediction and Analysis of P450s

The package Hmmer was used to predict P450s with Diamond 2.9.0 (e-value > 1 × 10^−5^) and annotate the target protein sequence. The reference P450 sequences for cluster analyses were downloaded from the website Fungal cytochrome P450 database (http://p450.riceblast.snu.ac.kr/index.php?a=view) (accessed on 15 July 2022). Totals of predicted 127 P450 proteins from *I. hispidus* NPCB_001 and several other Basidiomycetes selected from the fungal P450 database were clustered to perform phylogenetic tree analysis with precise classification. A maximum-likelihood tree was built by IQ-tree 2.2.3 with options as “-m MFP -bb 1000 -alrt 1000 -abayes -nt AUTO”.

### 2.8. Metabolites Analysis and Structural Evaluation

Fermentation products of mycelium and metabolites of fruiting bodies were used to analyze the small bioactive molecules of *I. hispidus*. Liquid fermentation of mycelium was performed in PDB at 200 rpm, 25 °C for 14 days. The fermentation product was extracted with ethyl acetate, concentrated, and quantified for high-resolution liquid chromatography-mass spectrometry (HR-LCMS) detection. Fresh fruit bodies were extracted with ethyl acetate, concentrated, and quantified for HRMS detection. The HRMS detection was carried out using AB Sciex TripleTOF 6600 mass spectrometer in both positive-ion and negative-ion modes. Molecular network analysis of HPLC-HRESIMS data of crude extract was performed using GNPS (https://gnps.ucsd.edu) (accessed on 17 September 2022) with default parameters. The network file based on positive-ion mode MS data can be found and Available online: https://gnps.ucsd.edu/ProteoSAFe/status.jsp?task=1c6ef68b5679494eb8017429b5ba9e77 (accessed on 1 September 2022). The molecular network was visualized by Cytoscape 3.9.1.

## 3. Results

### 3.1. Fungal Species Identity and Artificial Cultivation

The wild fruiting bodies of *I. hispidus* (Figure 1A) were collected from southern regions of Xinjiang province, China. The culturable mycelium (Figure 1B) was obtained by separating the fruiting body’s tissue. The sample was identified as *I. hispidus* by combining the morphological characteristics of the fruit bodies and the ITS sequence alignment (98.81% similarity to *Inonotus hispidus* clone SH2.107, Figure S1) of the mycelium, and was subsequently named *I. hispidus* NPCB_001. Given the economic potential of *I. hispidus’* medical capabilities, we attempted to produce it artificially and successfully obtained the fruiting body (Figure 1C). We now have the technology and equipment for large-scale artificial cultivation.

### 3.2. Genome Sequence, Assembly, and Annotation

**Figure 2:**
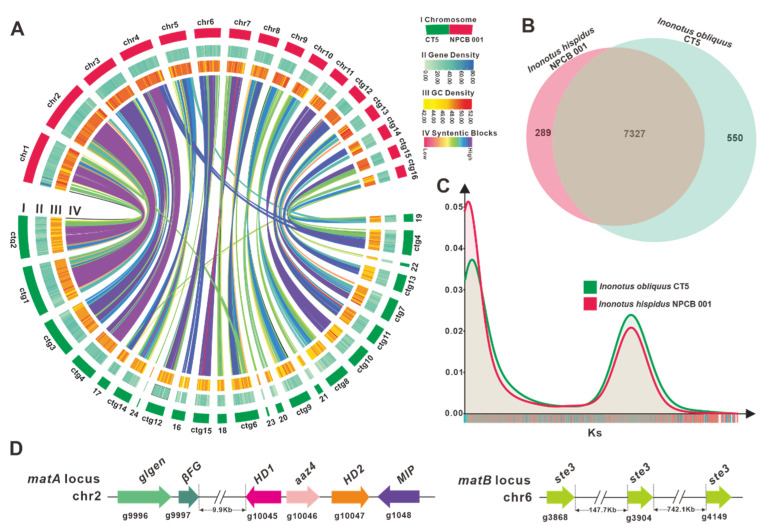
Genomic characterization, mating type loci, and comparative genomic analysis. (**A**) Genomic collinearity analysis between *I. hispidus* NPCB_001 and *I. obliquus* CT5. From the outside to the inside are I. Chromosome and Contigs; II&III. Gene density and GC density: the intensity of the color positively correlates with gene density; Ⅳ. Whole-genome collinearity analysis based on protein-coding genes: sequence similarity from low to high is indicated by red to purple. (**B**) Venn schematic of comparative genomes within *Inonotus* species. (**C**) Ks comparison within *Inonotus* species. (**D**) Structural diagram of the genes on the *matA* locus and *matB* locus of *I. obliquus*.

The genome size of *I. hispidus* NPCB_001 was determined to be 33.69 Mb based on the *k*-mer of the genome survey study (Table S1). A *K*-mer curve with two peaks and a 2-fold relationship in peak height revealed that the genome had heterozygosity of 0.992% (Figure S2), which indicated that *I. hispidus* NPCB_001 was a dikaryon. The genome of NPCB_001 was sequenced using a combination of the PacBio Nanopore and Illumina Hiseq sequencing platforms. A total of 8.12 Gb and 2.70 Gb clean data were generated from PacBio and Illumina sequencing platforms, respectively. A genome size of 34.02 Mb was built from totals of 5,729,964,000 bp of clean data, which comprised 11 pseudochromosomal molecules and six contigs (Figure 2A), with an N50 of 2,340,722 bp and 47.76% GC content (Figure 2A) (Tables S2 and S3). The illumina coverage ratio of 99.86% (Table S4) demonstrated that the genome of strain NPCB_001 was assembled with high quality.

There were 12,304 protein-coding genes predicted, with an average gene length of 1816.15 bp and a total of 78,769 exons (average length, 224.07 bp) and 660,465 introns (average length, 70.65 bp) in these coding genes (Table S5, File S1). Non-coding RNA was projected to include 14 rRNAs, 16 sRNAs, 105 tRNAs, and one snRNA (Table S6). A total of 7987 repeats with a total length of 1,100,314 bp were predicted, accounting for 3.23% of the whole genome, with the four scattered repeats SINE, LINE, LTR, and DNA transposons accounting for 0.00% (2), 0.06% (233), 2.20% (1384), and 1.51% (1024), respectively (Table S7). Genomic sequencing comparisons, assembly parameters, and quality metrics between *I. hispidus* and previously published *I. obliquus* [30] genomes emphasize the high-quality genome of the strain NPCB_001 (Table 1).

To archive the comprehensive protein-coding genes function annotation, 15,302 genes were subjected to sequence similarity analysis and motif similarity search based on nine public databases (Nr, Pfam, eggCOG, Uniprot, KEGG, GO, Pathway, Refseq, Interproscan) (Table S8). The Nr library annotation results found that 67.59% of the 10,580 annotated the genome of *Sanghuangporus baumii* and 22.44% matched the genome of *Fomitiporia mediterranea* MF3/22 (Figure S3). The classification of cellular components was the main group among the 5719 genes annotated by the functional classification of the GO database (Figure S4). Functional annotation based on the COG database identified 1051 genes, with the largest number of genes belonging to group I (lipid transport and metabolism) (Figure S5). According to the KEGG database, 3900 genes were identified as being involved in 5 types of pathways, with the largest number of genes involved in metabolic pathways (Figure S6). Domain-based motif search using the Pfam database identified 9523 genes, and the top 20 with the largest number are shown in Figure S7. These various perspectives and levels of annotation demonstrate the functional diversity of protein-coding genes from the strain NPCB_001.

### 3.3. Comparative Genomic Analysis within Inonotus Species

NCBI Taxonomy has documented 49 *Inonotus* species, and our team previously reported the genome of *I. obliquus* CT5 [30], the first genome of the *Inonotus* mushrooms. Collinearity analysis showed that practically all genomic regions of *I. hispidus* NPCB_001 shared synteny with the *I. obliquus* CT5 genome, and chr1, 2, 3, 4, and 11 of NPCB_001 exhibited high synteny to specific regions of the *I. obliquus* CT5 genome (Figure 2A). A total of 7327 orthologous groups were identified from the two species of *Inonotus*, and NPCB_001 contained relatively fewer unique orthologous groups (289) than that of CT5 (550) (Figure 2B). This finding verifies the discrepancy in genome size, with NPCB_001 having 34.02 Mb and CT5 having 38.18 Mb (Table 1).

To further understand the differences in the genomes of the species of *Inonotus*, a genome-wide duplication analysis based on synonymous mutation rates was performed. The consistent trends in the Ks curves of these strains revealed that they are all *Inonotus* species (Figure 2C). The obvious peaks in the Ks curves suggested that genome-doubling events occurred during the genomic evolution of the *Inonotus* species (Figure 2C). The higher KS peaks of *I. obliquus* CT5 indicated that it had undergone a larger-scale genome doubling event (Figure 2C), resulting in the larger-size genome of *I. obliquus* CT5 (Table 1).

### 3.4. Identification of the Mating Genes

Mushrooms, especially those formed by the phylum Basidiomycota fungi, contain a tetrapolar mating system composed of an A mating (*matA*) locus and a B mating (*matB*) locus. The locus *matA* mainly contains two homeodomain transcription factor-codon genes that control clamp-connection formation and nuclear pairing. The locus *matB* encodes multiple pheromone receptors (*ste3*) and pheromone precursors, which mainly regulate nuclear migration and clamp connection fusion [33,34]. The mating type genes in mushrooms are capable of controlling the process of hybridization and sexual reproduction. A comprehensive and in-depth understanding of the molecular genetic structure of the mating type system will aid in elucidating the regulation of mating type genes on fruit body development and solve the breeding-related scientific challenges faced in the development of the economically valuable mushroom industry [35,36].

For *I. hispidus* NPCB_001, the *matA* locus was located on chr2 by homology search using mitochondrial intermediate peptidase (mip) codon gene and HD1 of *I. obliquus* CT5, and the *matB* locus was located on chr6 by scanning with *ste3* from the strain CT5 as a probe. The *matA* locus comprises a MIP (g1048), three homeodomain transcription factor-codon genes (HD1, aαz4, and HD2), an unknown conserved fungal protein-codon gene (*βFG*, g9997), and a glycosyltransferase family 8 protein codon gene (*glgen*, g9996) (Figure 2D). HD1 (g1045) and HD2 (g1047) in *I. hispidus* NPCB_001 are two typical homeodomain transcription factor-codon genes in the *matA* locus with opposite transcription orientations. A-alpha Z4 (aαz4, g1046) represents a class of HD1 mating-type protein, which was first discovered in *Schizophyllum commune* H4-8 [37]. In contrast, the *matB* locus contains three unclustered *ste3* (g3868, g3904, and g4149) (Figure 2D) (Table S9). The analysis result, that the *matA* locus and the *matB* locus are not in the same contig, implies that the mating type of *I. hispidus* possesses a tetrapolar mating system. Overall, further research is required to better understand the genomic structure of the mating-type loci in *I. hispidus*.

### 3.5. Phylogenomic and Evolutionary Analysis

**Figure 3:**
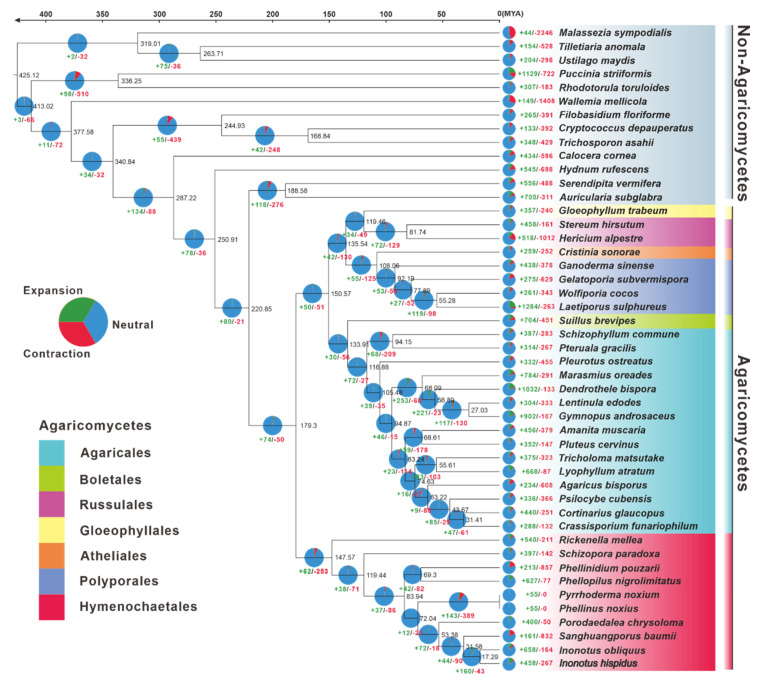
The evolutionary relationship and expanded and contracted gene families among *Inonotus* species and 45 representative Basidiomycetes. The maximum likelihood method credibility tree was inferred from 47 single-copy orthologous genes. All nodes received full bootstrap support. The divergence time is labeled as the mean crown age for each node, while the 95% highest posterior density is also given within the *Inonotus* clade. The black numbers at the branches indicate the corresponding divergence times in millions of years (MYA). The numbers of gene family expansion and contraction in each species are labeled with green and red symbols, respectively. The proportion of expansion and contraction in the genome of each species was displayed before its species name. The background color of each species of Agaricus indicates its corresponding order.

The genomes of 47 typical Basidiomycete mushrooms (Table S10) were utilized for phylogenomic evolutionary research to acquire insights into the evolutionary origins, taxonomic status, genome expansion, and contraction of *I. hispidus*. With complete bootstrap support, the phylogenomic tree constructed from an alignment of 67 single-copy orthologous genes from 91,926 orthogroups delineated evolutionary connections among the 47 species. The species from Agaricomycetes and Non-Agaricomycetes were phylogenetically separated at the species level. Hymenochaetales and non-Hymenochaetales of Agaricomycetes were diverged at a mean crown age of 179.30 Mya, with a 95% highest posterior density (HPD) of 97.55–256.83 Mya. *Inonotus* was estimated to emerge in a mean crown age of 31.58 Mya with a 95% HPD of 16.53–51.71 Mya, which had a closer phylogenetic relationship with *Sanghuangporus*. Of the species in *Inonotus*, *I. hispidus* and *I. obliquus* occurred in a mean crown age of 17.29 Mya with a 95% HPD of 8.95–28.70 Mya (Figure 3).

The gene family expansion happened more often than the gene family contraction in the evolutionary process of the 47 samples of Basidiomycota studied. Concerning *Inonotus*, 658 and 458 gene families had expanded in *I. hispidus* and *I. obliquus*, respectively, corresponding to 164 and 267 gene families being contracted. The *Inonotus* fungi had undergone more gene family expansion and less gene family contraction than *Sanghuangporus baumii* (Figure 3).

### 3.6. CAZyme Analysis and Synthesis of Polysaccharides

**Figure 4:**
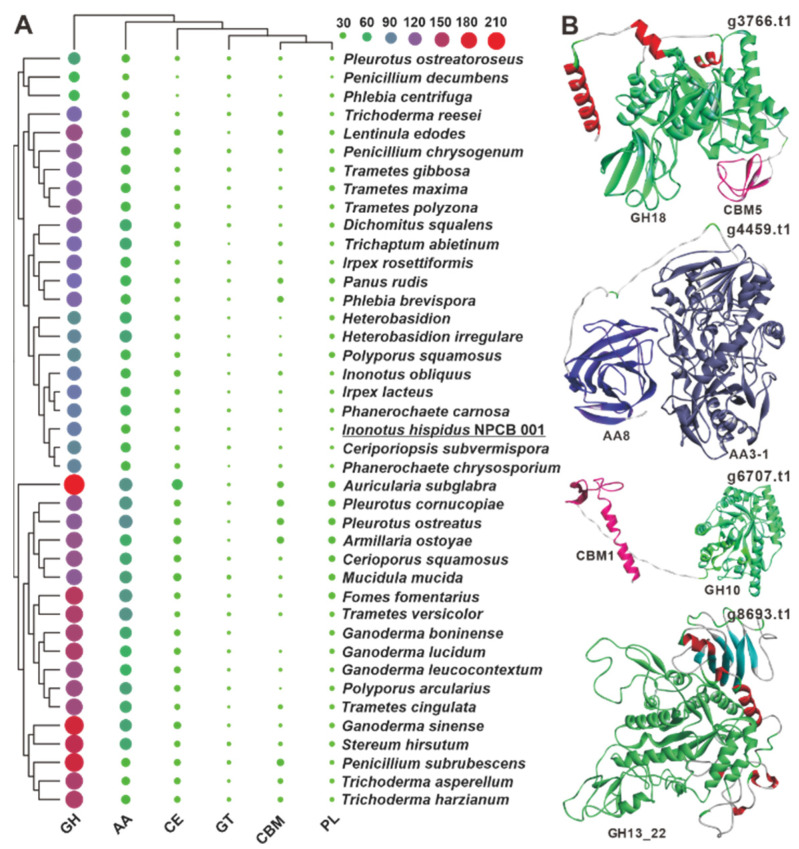
CAZymes analysis of *I. hispidus* and related white-rot fungi. (**A**) The sizes and colors (from green through purple to red) of circles in the bubble plot indicate the change in quantity, and the different colored triangles indicate different families. (**B**) The predicted structures of four bifunctional domain-containing CAZymes.

White-rot fungi are a group of fungi that effectively degrade lignocellulosic biomass, notably those of plant origin [38,39], and account for over 90% of wood-decaying stretcher fungi, degrading lignin and polysaccharides while leaving white or yellowish residues [39,40]. Although *I. hispidus* is a typical white-rot fungus, its CAZyme repertoire has not been investigated. Annotation of the predicted proteins of *I. hispidus* using the dbCAN2 CAZyme database revealed 151 CAZyme functional domains, including 95 glycoside hydrolases (GHs), 37 auxiliary activities (AAs), 11 carbohydrate esterases (CEs), three glycosyltransferases (GTs), three polysaccharide lyases (PLs), and two carbohydrate-binding modules (CBMs) in the strain NPCB_001 genome (Figure 4A, Table S11, File S2). The 151 functional domains are derived from 147 proteins, four of which, g3766, g4459, g6707, and g8693, contain bifunctional domains. Both g3766 and g6707 have a GH domain and a CBM domain, whereas g4459 and g8693 contain two AA domains (AA8, AA3-1) and two GH domains (GH13_22) (Figure 4B). Among the six classes of genes, the number of GHs is much higher than others and are mainly involved in the degradation of hemicellulose (GH10 and GH43), xyloglucan (GH16), celluloses (GH5 and GH12), and starch (GH15) (File S1). Regarding CAZyme distribution, *I. hispidus* is more similar to *Ceriporiopsis subvermispora* and *Phanerochaete chrysosporium* than to *I. obliquus*. When comparing the 39 analyzed white-rot fungi, it was found that the number and type of CAZymes of these white-rot fungi were not species-specific (Figure 4A).

*Inonotus hispidus* extracellular exopolysaccharide was discovered to protect the liver from acute alcoholic liver damage in mice [22]. Approximately 20 different enzymes involved in mushroom polysaccharide synthesis have been identified [41,42], including 1,3-glucan synthase (GLS), glucose phosphomutase (PGM), phosphomannose isomerase (PMI), glucokinase (GK), beta-glucan synthesis-associated protein (GSAP), phosphoglucose isomerase (PGI), UDP-glucose 4-epimerase (UGE), GDP-mannose dehydratase (GMD), phosphor-fructokinase (FPK), and UDP-xylose synthase (UXS). Screening relevant databases, 31 candidates (Table S12) for polysaccharide biosynthesis were identified, including 7 GMDs, 7 PGMs, 5 GSAPs, 2 UGEs, 2 GKs, 2 PMIs, and 2 GLSs, and only 1 FPK, PGI, FBPase, and UXS (Table S13).

### 3.7. The BGCs for Secondary Metabolite Analysis

**Figure 5:**
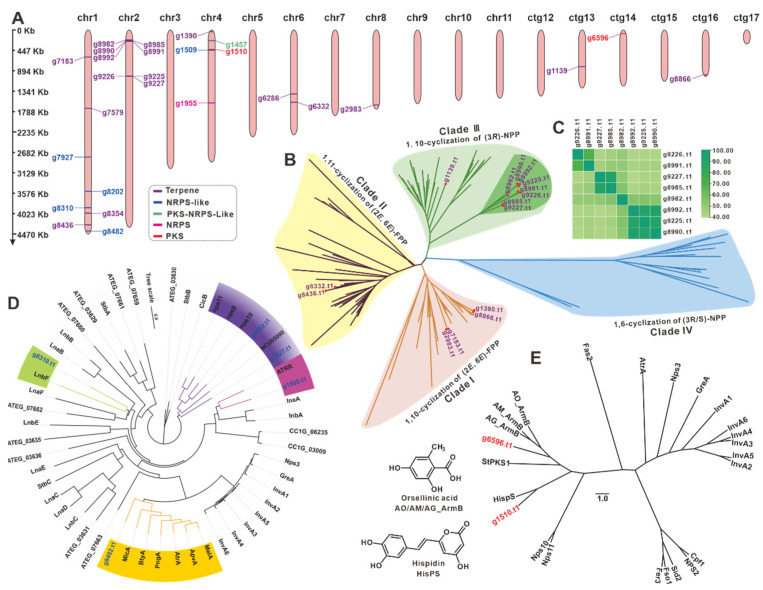
Analysis of genes involved in secondary metabolite biosynthesis. (**A**) Distribution of biosynthetic core genes for natural products on the chromosomes and contigs. Phylogenetic tree analysis sesquiterpene synthases (**B**), NRPS-likes (**D**), and PKSs (**E**) from NPCB _001 and their respective homologues. (**C**) The identity matrix of eight STSs.

The diversity of secondary metabolism in *I. obliquus* was explored by distinguishing the types of secondary metabolites based on the core enzymes engaged in the synthetic pathways. A total of 20 BGCs containing 27 core genes were predicted and distributed on five chromosomes (Chr1, 2, 4, 6, and 8) and three contigs (ctg13, 14, and 16) (Figure 5A) (Table 2). The 27 core genes include 18 terpene synthase-encoding genes, five genes for NRPS-like, two genes for PKS, and one gene each encoding NRPS and PKS-NRPS-like hybrid (Figure 5A).

There are 15 sesquiterpene synthases (STSs), one squalene synthase (g8354.t1), one oxidosqualene cyclase (g7579.t1), and a phytoene synthase (g6286.t1) among the 18 predicted terpenoid synthases. The evolutionary tree was constructed of the 15 STSs, and the identified STSs from Basidiomycete mushrooms [43] displayed four clear clades. Clade III had the most STSs (nine), followed by clade I with four, clade II with two, and clade IV without STS (Figure 5B, Table S14). Surprisingly, eight STSs in clade III formed a unique subcluster with high similarity (over 40% identities) (Figure 5C and Table S15). These STSs with high identities indicated the structural similarities of their catalytic products. Furthermore, six key genes (g10917, g7854, g1502, g245, g54, and g9473) of the MVP pathway upstream of terpenoid biosynthesis in *I. hispidus* were identified using the help of KEGG (Figure S8).

Cluster analysis of five NRPS-likes (Table S16) and identified NRPS-likes from fungi (Table S17) revealed five objects were clustered into four subclusters (Figure 5D). Both g8028 and g7297 in the same subcluster were predicted to have Adenylate-forming reductase activity. The protein encoded by g1959 was found to be similar to ATRR, an unusual glycine betaine reductase for choline biosynthesis in fungi [44]. The genes g8310 and g8482 may encode an MFS-type transporter [45] and a microperfuranone synthase [46], respectively. Cluster analysis of two PKSs (g6596.t1 and g1510.t1) (Table S18) and 22 identified PKSs from Basidiomycetes (Table S19) revealed that g6596.t1 and g1510.t1 were more closely linked to HispS (Figure 5E), a gene implicated in luciferin biosynthesis [47]. The PKS g9656.t1, on the other hand, was more closely linked to ArmBs [48], a family of orsellinic acid synthases. The BGC containing g1955.t1, the only NRPS from *I. hispidus*, was predicted to be involved in siderophore biosynthesis and is very similar to a BGC that exists in *I. obliquus* (Figure S9). Several key genes, including NRPS in these two BGCs, showed high homology with the identified BGC for siderophore in *Coprinopsis cinerea* [49] (Figure S9). The PKS-NRPS-like g1457.t1 is a rare hybrid enzyme in mushrooms, with its best and only search hit being HispS, at 38.60% identity [47].

### 3.8. Cytochrome P450 Family Analysis and Identification

**Figure 6:**
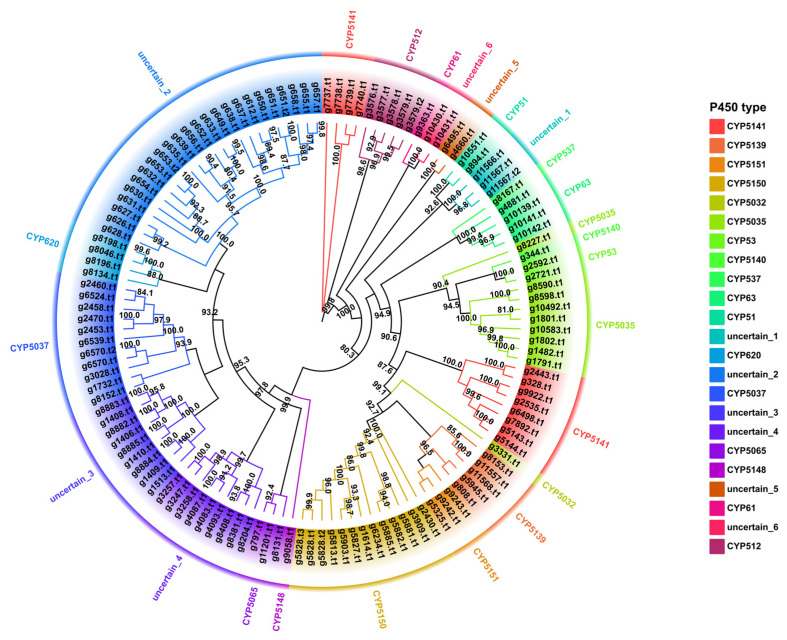
Maximum likelihood method tree of 127 P450s from the strain NPCB_001. Each P450 family is shown in a separate color, and the branch reliability value of not less than 50 is marked on the corresponding branch node.

The Cytochrome P450s (CYP450) family is a superfamily of thiol ferrous hemoglobin proteins that are widely involved in essential enzymes for fungal primary and secondary metabolic processes, including detoxification, exogenous degradation, and secondary product biosynthesis [50,51,52]. A total of 127 P450 proteins (Table S20) screened in the genome of the strain NPCB_001 were examined through clustering analysis with the representative Basidiomycete P450 proteins of the Fungal Cytochrome P450 Database. The clustering result offered a clear indication of the categorization of the P450s of strain NPCB_001 (Figure S10). Further cluster analysis classified the 127 P450s of *I. hispidus* NPCB_001 into 17 CYP families, five uncertain groups, and one completely unknown group. Among the 17 identified CYP families, CYP5150 had the most members with 13, followed by CYP5037 with 11, and the remainder of the families had no more than ten members (Figure 6). This feature is consistent with a pattern of enrichment of CYP5150 in Polyporale fungi [53]. The branch between two separate CYP families was defined as the uncertain group in evolutionary relatedness-based clustering analysis. A total of 51 CYP450s were clustered into six uncertain groups, accounting for 40.16% of all P450s. Among them, the uncertain_3 group contained the most members, with 25 CYP450s (Figure 6). The CPY450 members in uncertain groups suggest the existence of multiple new CYP450 types (families) in *I. hispidus*.

### 3.9. Identification and Difference of Metabolites from Fruiting Bodies and Mycelium

The fruiting body of *I. hispidus* is a classic folk medicine, containing diverse small molecule metabolites that endow a wide range of biological activities [10], although the chemical composition of the mycelium and their bioactivities are seldom described. In order to explore the variations in chemical composition and content between fruiting bodies and mycelium, metabolites obtained from fruiting bodies and mycelium fermentation in shaking flasks were utilized for quantitative HR-LCMS analysis and compared using GNPS online workflow.

**Figure 7:**
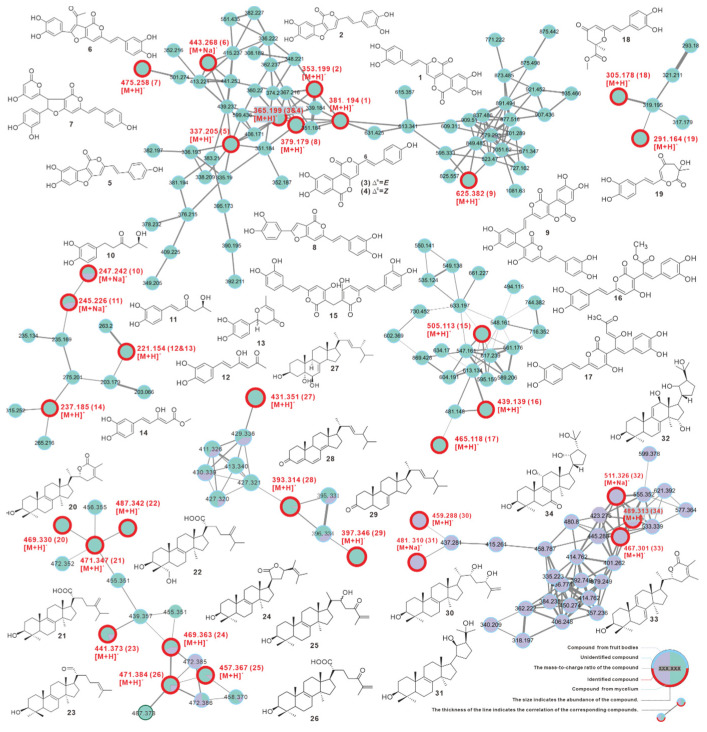
GNPS-based molecular network identification of metabolites from fruiting body and liquid culture of mycelium of *Inonotus hispidus*.

The visualized molecular network showed that mycelium produces more abundant chemical constituents, and most components are more closely related. In summary, the metabolites from mycelium and fruiting bodies differed widely in quantity and content (Figure 7, Figure S11).

Furthermore, a total of 34 compounds were identified by comparing their MS and MS2 data with reported literature values, including phelligridin D (**1**), phellibaumin A (**2**), phelligridin C (**3**), phelligridin C′ (**4**), 3′4′-dihydroxy-5-[11- hydroxyphenyl]-6,7-vinyl]-3,5-dioxafluoren-5-one (**5**), inoscavin C (**6**), hypholomine A (**7**), inoscavin E (**8**), inonoblin A (**9**), inonophenol A (**10**), inonophenol B (**11**), hispolon (**12**), hispinine (**13**), methyl 5-(3,4-dihydroxyphenyl)-3-hydroxypenta-2,4-dienoate (**14**), MBP (**15**), interfungin C (**16**), interfungin A (**17**), inonophenol C (**18**), inonotusin A (**19**), inotolactone B (**20**), eburicoic acid (**21**), hispindic acid B (**22**), 3β-hydroxy-lanosta-8,24-dien-21-al (**23**), inonotusol F (**24**), inonotusol G (**25**), inonotusane F (**26**), cerevisterol (**27**), 4,6,8(14),22(23)-tetraen-3-one-ergostane (**28**), 7(8),22(23)-dien-3-one-ergostane (**29**), inonotsutriol E (**30**), inonotsutriol A (**31**), inonotusane E (**32**), inotolactone A (**33**), and inonotusol E (**34**) (Figure 7 and Figure S12, Table 3). The identified chemicals are structurally divided into two groups, the styrylpyrones-based polyphenols (**1**–**19**) and the lanosterol-type triterpenoids (**20**–**34**), which correspond to the distinct clusters formed in the network (Figure 7, Table 3). Among these, 13 compounds were found for the first time from *I. hispidus*, including **7**–**9**, **16**–**17**, **24**–**26**, and **30**–**34**, and the majority of these compounds were initially described from the medicinal fungus Phellinus and other *Inonotus* species [54].

## 4. Discussion

### 4.1. Inonotus Hispidus and Sanghuang-like Fungi

*Sanghuang* is a well-known macrofungal medical herb in China, Japan, Korea, and other Asian countries. It has a long history of medicinal usage and health benefits [66,67,68], which is recorded in several Chinese medical classics, including the *Compendium of Materia Medica*. Indeed, *sanghuang* is recorded in various medical documents as a generic term for a group of medicinal macrofungi with specific biological activity and similar morphology, which is difficult to reconcile with modern species classification systems, seriously limiting modern medicinal research on *sanghuang*. Although the establishment of *Sanghuangporus* genus containing 15 species [32,69,70] has alleviated this dilemma to some degree, it has not been completely solved. Because of their similar morphology and medicinal properties to the *Sanghuangporus* species, several medicinal fungi from the genera *Inonotus* and *Phellinus*, such as *I. hispidus* [10] and *P. gilvus* [71], are still referred to as *sanghuang* in traditional medicine. Such fungi are more aptly known as sanghuang-like fungi. These valuable medicinal macrofungi should not be excluded from the Chinese medicinal fungus *sanghuang* because of the development of *Sanghuangporus* genus. The ability of genome sequencing to reliably correlate morphological traits to the genome enables the differentiation of sanghuang-like fungi from *Sanghuangporus* species, which is difficult to separate morphologically.

### 4.2. The Metabolites and Medicinal Properties of Inonotus Hispidus

The fruiting bodies of *I. hispidus* have traditionally been used as medicines for the treatment of indigestion, cancer, diabetes, and gastric diseases by the residents of Xinjiang province and Northeast China [3], and the biological activity studies based on monomeric compounds have revealed the mechanism of these pharmacological activities [10]. For example, the monomers identified in this work, such as phelligridin D (**1**) [72], phellibaumin A (**2**) [73], MBP (**15**) [59], inonotusin A (**19**) [15], and inotolactone B (**20**) [25] (Figure 7) have previously been proven to possess anti-cancer activity at various levels. A thorough survey revealed that the monomeric compounds with anti-cancer and antioxidant activity in *I. hispidus* are essentially polyphenols [10], which are also one of the distinguishing feature components of *I. hispidus*. Indeed, polyphenolic compounds with a styrylpyrone backbone are abundant in the genera *Phellinus* and *Inonotus* [54], as well as the recently established genus *Sanghuangporus* [74], reflecting the natural relationship of metabolites between sanghuang-like fungi and *Sanghuangporus* species. Furthermore, the molecular network constructed based on GNPS clearly displays the differences in the chemical composition of the fruiting bodies and mycelium of *I. hispidus.* This may serve as a precise guide for the targeted isolation of specific compounds (Figure 7 and Figure S10). The molecular network shows similarity clustering of related compounds and could facilitate identification of unknown molecules in the network (Figure 7 and Figure S10).

### 4.3. Genome Sequencing Helps Decipher Biosynthesis of Bioactive Ingredients in Medicinal Macrofungi

Genome sequencing of *G. lucidum* identified multiple biosynthetic genes necessary to produce ganoderic acids, which provided vital information to uncover the biosynthetic pathways of these essential medicinal components [27]. The genomic sequence of *A. cinnamomea* yielded critical candidate gene information to unravel antrocamphins biosynthesis in phase II clinical trials. Although polyphenols with a styrylpyrone moiety are a characteristic component of sanghuang-like fungi, the biosynthesis for these chemicals has received little attention [47,71,75]. Comprehensive analysis of the 20 gene clusters predicted in *I. hispidus* indicated that BGC12, with g1457 as a core gene, and BGC13, with g1510 as a core gene (Figure 5, Table 2), are connected to the biosynthesis of this class of polyphenols. HispS, which was able to convert Caffeic acid to Hispidin, is the best homologue of g1457.t1 and g1510.t1, with identities of 38.58% and 26.60%, respectively.

Secondary metabolite biosynthesis genes, especially post-modification genes, found in mushrooms tend to be scattered across chromosomes, similar to plants, rather than clustered like bacteria. The presence of high oxidation of polyphenolic compounds and triterpenoids in *I. hispidus* is thought to be related to the multiple P450s spread throughout the chromosomes, notably the over 50 P450 members of unidentified gene families (Figure 6, Table S20).

Basidiomycetes-derived sesquiterpenes are a class of important natural products with diverse structures and various activities, and sesquiterpene synthases are a class of critical genes for natural product biosynthesis [75]. According to an analysis of sequenced Basidiomycete genomes, each genome had at least ten sesquiterpene synthases. *I. obliquus*, a homology of *I. hispidus*, has more than 20 sesquiterpene synthases [30] and eight sesquiterpenoids [76], but *I. hispidus* only has 15 sesquiterpene synthases and one sesquiterpenoid xylaritriol [7], suggesting that the majority of the sesquiterpene synthases in the *I. hispidus* are inactive.

## 5. Conclusions

*Inonotus hispidus* is a well-known medicinal mushroom that exhibits anti-cancer and immunomodulatory activities, as well as a long history of usage as a medicinal fungal material with various health benefits. Here, for the first time, we provide the de novo assembled complete genome of *I. hispidus*. Chromosome-level assembly and functional annotation described in this study provide useful clues for subsequent gene functional research. According to comparative genomic analysis, the genus *Inonotus* has different gene compositions. Phylogenomic and evolutionary analysis of the genus *Inonotus* reveals evolutionary traits. The investigation of mate locus and CAZyme facilitates artificial cultivation. A thorough examination of secondary metabolite biosynthesis genes showed a wide range of biosynthetic potential. Further molecular network-based metabolite analysis revealed differences in chemical composition and concentration in fruiting bodies and mycelia. This work not only covers a vacuum in *I. hispidus* genetic information but also gives crucial insights into the biological aspects of the medicinal-edible fungus *I. hispidus*, such as growth characteristics and biosynthesis routes of bioactive components. A thorough grasp of I. hispidus’ genome will pave the way for its future application in pharmacological research and functional food development. In short, the genome sequencing of *I. hispidus* sheds light on the biosynthesis and medical applications of its metabolite.

## Figures and Tables

**Table 1:** Comparison of sequencing and assembly metrics, and genome quality of *I. hispidus* NPCB_001.

Species	*I. hispidus* NPCB_001	*I. obliquus* CT5
Sequencing technology	Illumina NovaSeq 6000	Illumina HiSeq 6000
Sequencing depth	230.0×	200.0×
Number of scaffolds	17	31
Total assembly length	34,017,109	38,061,412
largest length	4,469,123	4,380,421
Scaffold N50(bp)	2,340,722	1,971,511
Scaffold L50	5	7
GC content (%)	48.39	47.60
No. of proteins	12,304	12,525
Genome accession	GCA_024712875.1	GCA_023101745.1
Isolate information	Mycelium	Mycelium

**Table 2:** Putative BGCs responsible for secondary metabolites in the strain NPCB_001.

Cluster	Location	Start(bp)	Stop(bp)	Core Gene IDs	Core Gene Type
1	chr1	586,000	604,625	g7183.t1	terpene terpene
2	chr1	1,703,424	1,723,315	g7579.t1	
3	chr1	2,757,672	2,797,202	g7927.t1	NRPS-like
4	chr1	3,514,470	3,555,756	g8202.t1	NRPS-like
5	chr1	3,885,519	3,920,464	g8310.t1	NRPS-like
6	chr1	4,003,561	4,021,717	g8354.t1	terpene
7	chr1	4,255,843	4,277,160	g8436.t1	terpene
8	chr1	4,388,087	4,431,254	g8482.t1	NRPS-like
9	chr2	205,256	258,843	g8982.t1	terpene
				g8985.t1	
				g8990.t1	
				g8991.t1	
				g8992.t1	
10	chr2	999,219	1,027,559	g9225.t1	terpene
				g9226.t1	
				g9227.t1	
11	chr4	29,271	50,737	g1390.t1	terpene
12	chr4	213,291	257,392	g1457.t1	T1PKS- NRPS-like
13	chr4	407,063	453,131	g1509.t1	NRPS-like
				g1510.t1	T1PKS
14	chr4	1,576,159	1,624,146	g1955.t1	NRPS
15	chr6	1,395,705	1,411,230	g6286.t1	terpene
16	chr6	1,570,068	1,591,381	g6332.t1	terpene
17	chr8	1,624,840	1,646,078	g2983.t1	terpene
18	ctg13	785,200	806,403	g1139.t1	terpene
19	ctg14	61,058	108,404	g6596.t1	T1PKS
20	ctg16	977,869	999,335	g8866.t1	terpene

**Table 3:** The identified metabolites from the strain NPCB_001.

No	Source	Putative Metabolite	Molecular Formula	Adduct	*m*/*z*	Reference
1	fruiting body	phelligridin D	C_20_H_12_O_8_	[M + H]^+^	381.194	Li, et al. [55]
2	fruiting body	phellibaumin A	C_19_H_12_O_7_	[M + H]^+^	353.199	Li, et al. [55]
3	fruiting body	phelligridin C	C_20_H_12_O_7_	[M + H]^+^	365.199	Li, et al. [55]
4	fruiting body	phelligridin C′	C_20_H_12_O_7_	[M + H]^+^	365.199	Li, et al. [55]
5	fruiting body	3′4′-dihydroxy-5-[11- hydroxyphenyl]-6,7-vinyl]-3,5-dioxafluoren-5-one	C_19_H_12_O_6_	[M + H]^+^	337.205	Li, et al. [55]
6	fruiting body	inoscavin C	C_23_H_16_O_8_	[M + Na]^+^	443.268	Zan, et al. [56]
7 *	fruiting body	hypholomine A	C_26_H_18_O_9_	[M + H]^+^	475.258	Lee, et al. [54]
8 *	fruiting body	inoscavin E	C_21_H_14_O_7_	[M + H]^+^	379.179	Lee, et al. [57]
9 *	fruiting body	inonoblin A	C_33_H_20_O_13_	[M + H]^+^	625.382	Lee, et al. [58]
10	both	inonophenol A	C_12_H_16_O_4_	[M + Na]^+^	247.242	Kou, et al. [7]
11	both	inonophenol B	C_12_H_14_O_4_	[M + Na]^+^	245.226	Kou, et al. [7]
12	fruiting body	hispolon	C_12_H_12_O_4_	[M + H]^+^	221.154	Ali, N.A.A., et al. [4]
13	fruiting body	hispinine	C_12_H_12_O_4_	[M + H]^+^	221.154	Ren, et al. [8]
14	fruiting body	methyl 5-(3,4-dihydroxyphenyl)-3-hydroxypenta-2,4-dienoate	C_12_H_12_O_5_	[M + H]^+^	237.185	Yousfi, et al. [5]
15	fruiting body	MBP	C_27_H_20_O_10_	[M + H]^+^	505.113	Yang, et al. [59]
16 *	fruiting body	interfungin C	C_23_H_18_O_9_	[M + H]^+^	439.139	Lee, et al. [60]
17 *	fruiting body	interfungin A	C_25_H_20_O_9_	[M + H]^+^	465.118	Lee, et al. [60]
18	fruiting body	inonophenol C	C_16_H_16_O_6_	[M + H]^+^	305.178	Kou, et al. [7]
19	fruiting body	inonotusin A	C_15_H_14_O_6_	[M + H]^+^	291.164	Zan, et al. [56]
20	fruiting body	inotolactone B	C_31_H_48_O_3_	[M + H]^+^	469.330	Ying, et al. [25]
21	fruiting body	eburicoic acid	C_31_H_50_O_3_	[M + H]^+^	471.347	Yang, et al. [61]
22	fruiting body	hispindic acid B	C_31_H_50_O_4_	[M + H]^+^	487.342	Ren, et al. [8]
23	both	3β-hydroxy-lanosta-8,24-dien-21-al	C_30_H_48_O_2_	[M + H]^+^	441.373	Kou, et al. [7]
24 *	both	inonotusol F	C_31_H_48_O_3_	[M + H]^+^	469.368	Liu, et al. [62]
25 *	both	inonotusol G	C_30_H_48_O_3_	[M + H]^+^	457.367	Liu, et al. [62]
26 *	both	inonotusane F	C_30_H_46_O_4_	[M + H]^+^	471.384	Zhao, et al. [63]
27	fruiting body	cerevisterol	C_28_H_46_O_3_	[M + H]^+^	431.351	Kou, et al. [7]
28	both	4,6,8(14),22(23)-tetraen-3-one-ergostane	C_28_H_40_O	[M + H]^+^	393.314	Zan, et al. [56]
29	fruiting body	7(8),22(23)-dien-3-one-ergostane	C_28_H_40_O	[M + H]^+^	397.346	Zan, et al. [56]
30 *	mycelium	inonotsutriol E	C_30_H_50_O_3_	[M + H]^+^	459.288	Reiko Tanaka, et al. [64]
31 *	mycelium	inonotsutriol A	C_30_H_50_O_3_	[M + Na]^+^	481.310	Sayaka Taji, et al. [65]
32 *	mycelium	inonotusane E	C_30_H_50_O_3_	[M + Na]^+^	511.326	Zhao, et al. [63]
33 *	mycelium	inotolactone A	C_31_H_46_O_3_	[M + H]^+^	467.301	Ying, et al. [25]
34 *	mycelium	inonotusol E	C_30_H_48_O_5_	[M + H]^+^	489.313	Liu, et al. [62]

Both indicate the compound is driven from both the fruiting body and mycelium. An asterisk in the upper right-hand corner of the number indicates that the compound was first identified from *I. hispidu*.

## Data Availability

Not applicable.

## Supplementary Materials

The following supporting information can be downloaded at: https://www.mdpi.com/article/10.3390/jof8121245/s1, File S1: The information of 12,304 predicted protein-coding genes from *Inonotus hispidus* NPCB_001; File S2: The gene distribution of 41 white-rot fungi based on six major modules of CAZymes; Table S1: Genome size estimation for *I. hispidus* NPCB_001, Table S2: Statistics of Oxford Nanopore PromethION sequencing data volume of *I. hispidus* NPCB_001 genome, Table S3: Statistics of Illumina NovaSeq sequencing data volume information of *I. hispidus* NPCB_001 genome, Table S4: Statistics of Illumina NovaSeq sequencing data mapping of *I. hispidus* NPCB_001 genome, Table S5: Annotation Statistics of *I. hispidus* NPCB_001 coding gene, Table S6: Statistics of non-coding RNA annotation results in *I. hispidus* NPCB_ 001 genome, Table S7: Statistics of *I. hispidus* NPCB_001 repetitive sequence annotation results, Table S8: Statistics of *I. hispidus* NPCB_001 protein-coding gene annotation, Table S9: Candidate genes for mating type in *I. hispidus* NPCB_001 genome, Table S10: The source (URL) statistics for 47 representative Basidiomycetes used to phylogenetic analysis, Table S11: The source (URL) statistics for white-rot fungi used to analysis CAZymes, Table S12: The 31 candidate genes for polysaccharide biosynthesis screened in the database, Table S13: The candidate genes for polysaccharide biosynthesis in *I. hispidus* NPCB_001 genome, Table S14: Terpenoid biosynthesis related enzymes in *I. hispidus* NPCB_001, Table S15: Identify matrix of Eight STSs from *I. hispidus* NPCB_001, Table S16: NRPS-like biosynthesis related enzymes in *I. hispidus* NPCB_001 genome, Table S17: The 46 identified NRPS-likes from fungi used in cluster analysis, Table S18: Sequence homology analysis for two PKSs from *I. hispidus* NPCB_001, Table S19: The 22 identified PKS from Basidiomycota used in cluster analysis, Table S20: Identification of cytochrome P450 genes in *I. hispidus* NPCB_001 genome, Figure S1: ITS alignment of the strain NPCB_001, Figure S2: *K*-mer Depth and *K*-mer Species Frequency Distribution Plot, Figure S3: Species distribution map of Nr database alignment to sequences, Figure S4: Statistical map of functional annotation classification based on Gene Ontology database, Figure S5: Statistical Chart of COG Functional Annotated Classification, Figure S6: KEGG Pathway Functional Classification Chart, Figure S7: Domain annotation based on the Pfam database, Figure S8: Annotation of the terpenoid backbone biosynthesis of NPCB_001 using KAAS, Figure S9: Functional analysis of NRPS and comparative analysis of the BGCs involved, Figure S10: P450s Cluster analysis of the strain *I. hispidus* NPCB_001 and other Basidiomycete, Figure S11: Molecular network analysis of metabolites from the mycelium and fruiting bodies of the strain *I. hispidus* NPCB_001, Figure S12: The LC-ESI-HRMS and LC-ESI-HRMS/MS spectrums of isolates from the strain *I. hispidus* NPCB_001. Refs [49,77,78,79] are cited in supplementary materials.

## References

[1] Piątek M., Wojewoda W. (2000). *Inonotus hispidus* (Bull.: Fr.) Karst. Atlas of the Geographical Distribution of Fungi in Poland.

[2] Song F., Su D., Keyhani N.O., Wang C., Shen L., Qiu J. (2022). Influence of selenium on the mycelia of the shaggy bracket fungus, *Inonotus hispidus*. J. Sci. Food Agric..

[3] Zan L.-F., Bao H.-Y. (2011). Progress in Inonotus hispidus research. Acta Edulis Fungi.

[4] Ali N.A.A., Jansen R., Pilgrim H., Liberra K., Lindequist U. (1996). Hispolon, a yellow pigment from *Inonotus hispidus*. Phytochemistry.

[5] Yousfi M., Djeridane A., Bombarda I., Chahrazed H., Duhem B., Gaydou E.M. (2009). Isolation and Characterization of a New Hispolone Derivative from Antioxidant Extracts of *Pistacia atlantica*. Phytother. Res..

[6] Li Z., Bao H. (2022). Deciphering key regulators of *Inonotus hispidus* petroleum ether extract involved in anti-tumor through whole transcriptome and proteome analysis in H22 tumor-bearing mice model. J. Ethnopharmacol..

[7] Kou R.W., Du S.T., Xia B., Zhang Q., Yin X., Gao J.M. (2021). Phenolic and Steroidal Metabolites from the Cultivated Edible *Inonotus hispidus* Mushroom and Their Bioactivities. J. Agric. Food Chem..

[8] Ren Q., Lu X.-Y., Han J.-X., Aisa H.A., Yuan T. (2017). Triterpenoids and phenolics from the fruiting bodies of *Inonotus hispidus* and their activations of melanogenesis and tyrosinase. Chin. Chem. Lett..

[9] Perrin P.W., Towers G.H.N. (1973). Hispidin biosynthesis in cultures of *Polyporus hispidus*. Phytochemistry.

[10] Wang Z., Feng X., Liu C., Gao J., Qi J. (2022). Diverse Metabolites and Pharmacological Effects from the Basidiomycetes *Inonotus hispidus*. Antibiotics.

[11] Kahlos K., Hiltunen R., v Schantz M. (1984). 3β-Hydroxy-lanosta-8,24-dien-21-al, a New Triterpene from *Inontus obliquus*. Planta Med..

[12] Hou R., Liu X., Xiang K., Chen L., Wu X., Lin W., Zheng M., Fu J. (2019). Characterization of the physicochemical properties and extraction optimization of natural melanin from *Inonotus hispidus* mushroom. Food Chem..

[13] Li Z., Bao H. (2022). Anti-tumor effect of *Inonotus hispidus* petroleum ether extract in H22 tumor-bearing mice and analysis its mechanism by untargeted metabonomic. J. Ethnopharmacol..

[14] Yang S., Bao H., Wang H., Li Q. (2019). Anti-tumour Effect and Pharmacokinetics of an Active Ingredient Isolated from *Inonotus hispidus*. Biol. Pharm. Bull..

[15] Zan L.-F., Qin J.-C., Zhang Y.-M., Yao Y.-H., Bao H.-Y., Li X. (2011). Antioxidant Hispidin Derivatives from Medicinal Mushroom *Inonotus hispidus*. Chem. Pharm. Bull..

[16] Angelini P., Girometta C., Tirillini B., Moretti S., Covino S., Cipriani M., D’Ellena E., Angeles G., Federici E., Savino E. (2019). A comparative study of the antimicrobial and antioxidant activities of *Inonotus hispidus* fruit and their mycelia extracts. Int. J. Food Prop..

[17] Min T., Ye D., Yang Y., Xie M.-L., Wang S.-M., Chen C.-B., Wang H., Li Y. (2021). α-glucosidase Inhibition and Antioxidant Activities of Different Polar Extracts of *Inonotus hispidus*. Edible Fungi China.

[18] Shomali N., Onar O., Alkan T., Demirtaş N., Akata I., Yildirim Ö. (2019). Investigation of the Polyphenol Composition, Biological Activities, and Detoxification Properties of Some Medicinal Mushrooms from Turkey. Turk J. Pharm. Sci..

[19] Wang L., Hou Y. (2011). Determination of trace elements in anti-influenza virus mushrooms. Biol. Trace Elem. Res..

[20] Ali N.A.A., Mothana R.A.A., Lesnau A., Pilgrim H., Lindequist U. (2003). Antiviral activity of *Inonotus hispidus*. Fitoterapia.

[21] Gruendemann C., Arnhold M., Meier S., Baecker C., Garcia-Kaeufer M., Grunewald F., Steinborn C., Klemd A.M., Wille R., Huber R. (2016). Effects of *Inonotus hispidus* Extracts and Compounds on Human Immunocompetent Cells. Planta Med..

[22] Liu X., Hou R., Yan J., Xu K., Wu X., Lin W., Zheng M., Fu J. (2019). Purification and characterization of *Inonotus hispidus* exopolysaccharide and its protective effect on acute alcoholic liver injury in mice. Int. J. Biol. Macromol..

[23] Benarous K., Djeridane A., Kameli A., Yousfi M. (2013). Inhibition of Candida rugosa Lipase by Secondary Metabolites Extracts of Three Algerian Plants and their Antioxydant Activities. Curr. Enzym. Inhib..

[24] Benarous K., Bombarda I., Iriepa I., Moraleda I., Gaetan H., Linani A., Tahri D., Sebaa M., Yousfi M. (2015). Harmaline and hispidin from *Peganum harmala* and *Inonotus hispidus* with binding affinity to Candida rugosa lipase: In silico and in vitro studies. Bioorg. Chem..

[25] Ying Y.-M., Zhang L.-Y., Zhang X., Bai H.-B., Liang D.-E., Ma L.-F., Shan W.-G., Zhan Z.-J. (2014). Terpenoids with alpha-glucosidase inhibitory activity from the submerged culture of *Inonotus obliquus*. Phytochemistry.

[26] Jiang N., Hu S., Peng B., Li Z., Yuan X., Xiao S., Fu Y. (2021). Genome of Ganoderma Species Provides Insights Into the Evolution, Conifers Substrate Utilization, and Terpene Synthesis for *Ganoderma tsugae*. Front. Microbiol..

[27] Chen S., Xu J., Liu C., Zhu Y., Nelson D.R., Zhou S., Li C., Wang L., Guo X., Sun Y. (2012). Genome sequence of the model medicinal mushroom *Ganoderma lucidum*. Nat. Commun..

[28] Lu M.Y., Fan W.L., Wang W.F., Chen T., Tang Y.C., Chu F.H., Chang T.T., Wang S.Y., Li M.Y., Chen Y.H. (2014). Genomic and transcriptomic analyses of the medicinal fungus Antrodia cinnamomea for its metabolite biosynthesis and sexual development. Proc. Natl. Acad. Sci. USA.

[29] Gong W., Wang Y., Xie C., Zhou Y., Zhu Z., Peng Y. (2020). Whole genome sequence of an edible and medicinal mushroom, *Hericium erinaceus* (Basidiomycota, Fungi). Genomics.

[30] Duan Y., Han H., Qi J., Gao J.-M., Xu Z., Wang P., Zhang J., Liu C. (2022). Genome sequencing of *Inonotus obliquus* reveals insights into candidate genes involved in secondary metabolite biosynthesis. BMC Genom..

[31] Dong W.-G., Wang Z.-X., Feng X.-L., Zhang R.-Q., Shen D.-Y., Du S., Gao J.-M., Qi J. (2022). Chromosome-Level Genome Sequences, Comparative Genomic Analyses, and Secondary-Metabolite Biosynthesis Evaluation of the Medicinal Edible Mushroom *Laetiporus sulphureus*. Microbiol. Spectr..

[32] Wu S.H., Chang C.C., Wei C.L., Jiang G.Z., Cui B.K. (2019). *Sanghuangporus toxicodendri* sp. nov. (Hymenochaetales, Basidiomycota) from China. MycoKeys.

[33] Kües U., Casselton L.A. (1992). Homeodomains and regulation of sexual development in *basidiomycetes*. Trends Genet..

[34] Casselton L.A., Olesnicky N.S. (1998). Molecular Genetics of Mating Recognition in Basidiomycete Fungi. Microbiol. Mol. Biol. Rev..

[35] Chen C.-L., Li W.-C., Chuang Y.-C., Liu H.-C., Huang C.-H., Lo K.-Y., Chen C.-Y., Chang F.-M., Chang G.-A., Lin Y.-L. (2022). Sexual Crossing, Chromosome-Level Genome Sequences, and Comparative Genomic Analyses for the Medicinal Mushroom *Taiwanofungus Camphoratus* (Syn. *Antrodia Cinnamomea, Antrodia Camphorata*). Microbiol. Spectr..

[36] Wang G., Wang Y., Chen L., Wang H., Guo L., Zhou X., Dou M., Wang B., Lin J., Liu L. (2021). Genetic structure and evolutionary diversity of mating-type (MAT) loci in *Hypsizygus marmoreus*. IMA Fungus.

[37] Ohm R.A., de Jong J.F., Lugones L.G., Aerts A., Kothe E., Stajich J.E., de Vries R.P., Record E., Levasseur A., Baker S.E. (2010). Genome sequence of the model mushroom *Schizophyllum commune*. Nat. Biotech..

[38] Eriksson K.-E.L., Blanchette R.A., Ander P. (1990). Morphological Aspects of Wood Degradation by Fungi and Bacteria. Microbial and Enzymatic Degradation of Wood and Wood Components.

[39] Sista Kameshwar A.K., Qin W. (2018). Comparative study of genome-wide plant biomass-degrading CAZymes in white rot, brown rot and soft rot fungi. Mycology.

[40] Rytioja J., Hildén K., Yuzon J., Hatakka A., Vries R.P.D., Mäkelä M.R. (2014). Plant-Polysaccharide-Degrading Enzymes from Basidiomycetes. Microbiol. Mol. Biol. Rev..

[41] Peng L., Qiao S., Xu Z., Guan F., Ding Z., Gu Z., Zhang L., Shi G. (2015). Effects of culture conditions on monosaccharide composition of *Ganoderma lucidum* exopolysaccharide and on activities of related enzymes. Carbohyd. Polym..

[42] Zhang N., Tang Z., Zhang J., Li X., Yang Z., Yang C., Zhang Z., Huang Z. (2019). Comparative transcriptome analysis reveals the genetic basis underlying the biosynthesis of polysaccharides in *Hericium erinaceus*. Bot. Stud..

[43] Wu J., Yang X., Duan Y., Wang P., Qi J., Gao J.-M., Liu C. (2022). Biosynthesis of Sesquiterpenes in Basidiomycetes: A Review. J. Fungi.

[44] Hai Y., Huang A.M., Tang Y. (2019). Structure-guided function discovery of an NRPS-like glycine betaine reductase for choline biosynthesis in fungi. Proc. Natl. Acad. Sci. USA.

[45] Forseth R.R., Amaike S., Schwenk D., Affeldt K.J., Hoffmeister D., Schroeder F.C., Keller N.P. (2013). Homologous NRPS-like Gene Clusters Mediate Redundant Small-Molecule Biosynthesis in *Aspergillus flavus*. Angew. Chem. Int. Edit..

[46] Yeh H.-H., Chiang Y.-M., Entwistle R., Ahuja M., Lee K.-H., Bruno K.S., Wu T.-K., Oakley B.R., Wang C.C.C. (2012). Molecular genetic analysis reveals that a nonribosomal peptide synthetase-like (NRPS-like) gene in *Aspergillus nidulans* is responsible for microperfuranone biosynthesis. Appl. Microbiol. Biotechnol..

[47] Kotlobay A.A., Sarkisyan K.S., Mokrushina Y.A., Marcet-Houben M., Serebrovskaya E.O., Markina N.M., Gonzalez Somermeyer L., Gorokhovatsky A.Y., Vvedensky A., Purtov K.V. (2018). Genetically encodable bioluminescent system from fungi. Proc. Natl. Acad. Sci. USA.

[48] Engels B., Heinig U., Grothe T., Stadler M., Jennewein S. (2011). Cloning and Characterization of an Armillaria gallica cDNA Encoding Protoilludene Synthase, Which Catalyzes the First Committed Step in the Synthesis of Antimicrobial Melleolides *. J. Biol. Chem..

[49] Tsunematsu Y., Takanishi J., Asai S., Masuya T., Nakazawa T., Watanabe K. (2019). Genomic Mushroom Hunting Decrypts Coprinoferrin, A Siderophore Secondary Metabolite Vital to Fungal Cell Development. Org. Lett..

[50] Bhattacharyya S., Sinha K., Sil C.P. (2014). Cytochrome P450s: Mechanisms and Biological Implications in Drug Metabolism and its Interaction with Oxidative Stress. Curr. Drug Metab..

[51] Zhang X., Guo J., Cheng F., Li S. (2021). Cytochrome P450 enzymes in fungal natural product biosynthesis. Nat. Prod. Rep..

[52] Durairaj P., Hur J.-S., Yun H. (2016). Versatile biocatalysis of fungal cytochrome P450 monooxygenases. Micro. Cell Fact..

[53] Fessner N.D., Nelson D.R., Glieder A. (2021). Evolution and enrichment of CYP5035 in Polyporales: Functionality of an understudied P450 family. Appl. Microbiol. Biotechnol..

[54] Lee I.-K., Yun B.-S. (2011). Styrylpyrone-class compounds from medicinal fungi *Phellinus* and *Inonotus* spp., and their medicinal importance. J. Antibiot..

[55] Qing-Jie L. (2017). Study on the active substances and quality standards of Sanghuang fungus. Ph.D. Thesis.

[56] Lifeng Z. (2012). Studies on the Chemical Constituents and Pharmacological Activities of *Inonotus Hispidus* and *Fomitiporia Ellipsoidea*. Ph.D. Thesis.

[57] Lee I.K., Kim Y.S., Seok S.J., Yun B.S. (2007). Inoscavin E, a Free Radical Scavenger from the Fruiting Bodies of *Inonotus xeranticus*. J. Antibiot..

[58] Lee I.K., Kim Y.S., Jang Y.W., Jung J.Y., Yun B.S. (2007). New antioxidant polyphenols from the medicinal mushroom *Inonotus obliquus*. Bioorg. Med. Chem. Lett..

[59] Yang S.-D., Bao H.-Y., Wang H. (2019). Chemical components and anti-tumour compounds from *Inonotus hispidus*. Mycosystema.

[60] Lee I.K., Yun B.S. (2007). Highly oxygenated and unsaturated metabolites providing a diversity of hispidin class antioxidants in the medicinal mushrooms *Inonotus* and *Phellinus*. Bioorg. Med. Chem..

[61] Xiu-Zhen Y. (2008). Studies on the Chemical Constituents of *Xanthochrous Hispidus*. Master’s Thesis.

[62] Liu C., Zhao C., Pan H.H., Kang J., Yu X.T., Wang H.Q., Li B.M., Xie Y.Z., Chen R.Y. (2014). Chemical constituents from *Inonotus obliquus* and their biological activities. J. Nat. Prod..

[63] Zhao F., Xia G., Chen L., Zhao J., Xie Z., Qiu F., Han G. (2016). Chemical constituents from *Inonotus obliquus* and their antitumor activities. J. Nat. Med..

[64] Tanaka R., Toyoshima M., Yamada T. (2011). New lanostane-type triterpenoids, inonotsutriols D, and E, from *Inonotus obliquus*. Phytochem. Lett..

[65] Taji S., Yamada T., Tanaka R. (2008). Three New Lanostane Triterpenoids, Inonotsutriols A, B, and C, from *Inonotus obliquus*. Helv. Chim. Acta.

[66] Chen W., Tan H., Liu Q., Zheng X., Zhang H., Liu Y., Xu L. (2019). A Review: The Bioactivities and Pharmacological Applications of *Phellinus linteus*. Molecules.

[67] He P., Zhang Y., Li N. (2021). The phytochemistry and pharmacology of medicinal fungi of the genus *Phellinus*: A review. Food Funct..

[68] Chen H., Tian T., Miao H., Zhao Y.-Y. (2016). Traditional uses, fermentation, phytochemistry and pharmacology of *Phellinus linteus*: A review. Fitoterapia.

[69] Zhu L., Song J., Zhou J.-L., Si J., Cui B.-K. (2019). Species Diversity, Phylogeny, Divergence Time, and Biogeography of the Genus *Sanghuangporus* (Basidiomycota). Front. Microbiol..

[70] Wu S., Dai Y., Hattori T., Yu T., Wang D., Parmasto É.K., Zhang H., Shi S. (2012). Species clarification for the medicinally valuable ‘sanghuang’ mushroom. Bot. Stud..

[71] Huo J., Zhong S., Du X., Cao Y., Wang W., Sun Y., Tian Y., Zhu J., Chen J., Xuan L. (2020). Whole-genome sequence of *Phellinus gilvus* (mulberry Sanghuang) reveals its unique medicinal values. J. Adv. Res..

[72] Suabjakyong P., Saiki R., Van Griensven L.J.L.D., Higashi K., Nishimura K., Igarashi K., Toida T. (2015). Polyphenol Extract from *Phellinus igniarius* Protects against Acrolein Toxicity In Vitro and Provides Protection in a Mouse Stroke Model. PLoS ONE.

[73] Wu C.-S., Lin Z.-M., Wang L.-N., Guo D.-X., Wang S.-Q., Liu Y.-Q., Yuan H.-Q., Lou H.-X. (2011). Phenolic compounds with NF-κB inhibitory effects from the fungus *Phellinus baumii*. Bio. Med. Chem. Lett..

[74] Zhang J.-J., Chen B.-S., Dai H.-Q., Ren J.-W., Zhou L.-W., Wu S.-H., Liu H.-W. (2021). Sesquiterpenes and polyphenols with glucose-uptake stimulatory and antioxidant activities from the medicinal mushroom *Sanghuangporus sanghuang*. Chin. J. Nat. Med..

[75] Zhang H., Chen R., Zhang J., Bu Q., Wang W., Liu Y., Li Q., Guo Y., Zhang L., Yang Y. (2019). The integration of metabolome and proteome reveals bioactive polyphenols and hispidin in ARTP mutagenized *Phellinus baumii*. Sci. Rep..

[76] Duan Y., Sui D., Wang L., Zhang X., Wang C., Liu C. (2021). Research Progress of Small Molecule Chemical Components and Pharmacological Values of *Inonotus obliquus*. J. Fungal Res..

[77] Gilchrist C.L.M., Chooi Y.-H. (2021). Clinker & clustermap.js: Automatic generation of gene cluster comparison figures. Bioinformatics.

[78] Nakamura T., Yamada K.D., Tomii K., Katoh K. (2018). Parallelization of MAFFT for large-scale multiple sequence alignments. Bioinformatics.

[79] Minh B.Q., Schmidt H.A., Chernomor O., Schrempf D., Woodhams M.D., von Haeseler A., Lanfear R. (2020). IQ-TREE 2: New Models and Efficient Methods for Phylogenetic Inference in the Genomic Era. Mol. Biol. Evol..

