# Supplementary material for: Genomic and Metabolomic Analyses of the Medicinal Fungus Inonotus hispidus for Its Metabolite’s Biosynthesis and Medicinal Application
Source: J Fungi (Basel). 2022 Nov 25;8(12):1245. doi: 10.3390/jof8121245 (PMC9787987; doi:10.3390/jof8121245)
Supplement: Supplementary file 1 [file jof-08-01245-s001.zip › Supplemental material.pdf]

Genomic and metabolomic analyses of the medicinal fungus *Inonotus hispidus* for its metabolite's biosynthesis and medicinal application

Rui-qi Zhang[1], Xi-long Feng[1], Zhen-xin Wang[1], Tian-chen Xie[1], Yingce Duan[2], Chengwei Liu[2] Jin-ming Gao[1] and Jianzhao Qi [1*]

1 Shaanxi Key Laboratory of Natural Products & Chemical Biology, College of Chemistry & Pharmacy, Northwest A&F University, Yangling 712100, China

2 Key Laboratory for Enzyme and Enzyme-Like Material Engineering of Heilongjiang, College of Life Science, Northeast Forestry University, Harbin 150040, China

* Correspondence: qjz@nwafu.edu.cn

Content

Table S1. Genome size estimation for *I. hispidus* NPCB_001...1
Table S2. Statistics of Oxford Nanopore PromethION sequencing data volume of *I. hispidus* NPCB_001 genome..2
Table S3. Statistics of Illumina NovaSeq sequencing data volume information of *I. hispidus* NPCB_001 genome..3
Table S4. Statistics of Illumina NovaSeq sequencing data mapping of *I. hispidus* NPCB_001 genome...4
Table S5. Annotation Statistics of *I. hispidus* NPCB_001 coding gene...5
Table S6. Statistics of non-coding RNA annotation results in *I. hispidus* NPCB_ 001 genome. 6
Table S7. Statistics of *I. hispidus* NPCB_001 repetitive sequence annotation results. 7
Table S8. Statistics of *I. hispidus* NPCB_001 protein-coding gene annotation. 8
Table S9. Candidate genes for mating type in I. hispidus NPCB_001 genome. 9
Table S10. The source (URL) statistics for 47 representative Basidiomycetes used to phylogenetic analysis. 10
Table S11. The source (URL) statistics for white-rot fungi used to analysis CAZymes.........12
Table S12. The 31 candidate genes for polysaccharide biosynthesis screened in the database. 14
Table S13. The candidate genes for polysaccharide biosynthesis in *I. hispidus* NPCB_001 genome...15
Table S14. Terpenoid biosynthesis related enzymes in *I. hispidus* NPCB_001. 17
Table S15. Identify matrix of Eight STSs from *I. hispidus* NPCB_001..................................18
Table S16. NRPS-like biosynthesis related enzymes in *I. hispidus* NPCB_001 genome. 19
Table S17. The 46 identified NRPS-likes from fungi used in cluster analysis........................20
Table S18. Sequence homology analysis for two PKSs from *I. hispidus* NPCB_001. 21
Table S19. The 22 identified PKS from Basidiomycetes used in cluster analysis. 22
Table S20. Identification of cytochrome P450 genes in *I. hispidus* NPCB_001 genome. 23
Figure S1. ITS alignment of the strain NPCB_001...27
Figure S2. *K*-mer Depth and *K*-mer Species Frequency Distribution Plot.............................28
Figure S3. Species distribution map of Nr database alignment to sequences. 29
Figure S4. Statistical map of functional annotation classification based on Gene Ontology database..30
Figure S5. Statistical Chart of COG Functional Annotated Classification. 31
Figure S6. KEGG Pathway Functional Classification Chart. 32
Figure S7. Domain annotation based on the Pfam database. 33
Figure S8. Annotation of the terpenoid backbone biosynthesis of NPCB_001 using KAAS. 34
Figure S9. Functional analysis of NRPS and comparative analysis of the BGCs involved....35
Figure S10. P450s Cluster analysis of the strain *I. hispidus* NPCB_001 and other Basidiomycetes. 36
Figure S11. Molecular network analysis of metabolites from the mycelium and fruiting bodies of the strain *I. hispidus* NPCB_001. 37
Figure S12. The LC-ESI-HRMS and LC-ESI-HRMS/MS spectrums of isolates from the strain *I. hispidus* NPCB_001...40
Reference 41

Table S1. Genome size estimation for *I. hispidus* NPCB_001.

**Item**	**Number**
Heterozygosity	0.922%
Genome Haploid Length	33,688,229 bp
Genome Repeat Length	8,699,429 bp
Genome Unique Length	24,988,801 bp
Model Fit	96.598%
Read Error Rate	0.051%

Genome Haploid Length is genome size, **Genome Repeat Length** is the length of repeat sequences, **Genome Unique Length** is the length of non-repeated sequences, **Read Error Rate** is read error rate.

Table S2. Statistics of Oxford Nanopore PromethION sequencing data volume of *I. hispidus* NPCB_001 genome.

Rank	Flag	TotalBase	TotalReads	MaxLen	AvgLen	N50	L50	N90	L90	meanQ
0	all	8,044,663,434	1,481,781	280,975	5,429.05	23,187	98,450	1,803	689,957	11.30
0	pass	7,813,963,986	1,427,135	173,179	5,475.28	23,402	95,157	1,815	662,852	11.49
0	fail	230,699,448	54,646	280,975	4,221.7	15,682	3,492	1,500	27,513	6.27
5000	all	6,097,918,399	288,273	280,975	21,153.27	32,182	62,976	9,053	196,294	11.39
5000	pass	5,938,786,064	279,676	173,179	21,234.52	32,292	61,191	9,102	190,325	11.55
5000	fail	159,132,335	8,597	280,975	18,510.21	28,266	1,813	7,776	6,021	6.28
10000	all	5,380,194,634	184,944	280,975	29,090.93	35,650	52,381	15,265	141,597	11.45
10000	pass	5,246,124,614	179,965	173,179	29,150.8	35,725	50,995	15,305	137,759	11.59
10000	fail	134,070,020	4,979	280,975	26,927.09	32,372	1,397	14,037	3,850	6.27
50000	all	1,420,567,847	22,174	280,975	64,064.57	62,677	9,379	51,988	19,388	11.51
50000	pass	1,390,757,850	21,712	173,179	64,054.8	62,705	9,187	51,984	18,984	11.62
50000	fail	29,809,997	462	280,975	64,523.8	61,736	193	52,232	404	6.26
100000	all	57,844,930	510	280,975	113,421.43	109,354	233	101,465	453	10.85
100000	pass	55,687,999	494	173,179	112,728.74	109,273	227	101,426	439	11.02
100000	fail	2,156,931	16	280,975	134,808.18	130,776	7	104,804	14	5.78

Rank is the gradient of data length, **0** is that all data; **Flag** is data type, **all** is all sequencing data, **pass** is effective sequencing data, **fail** is filtration data; **TotalBase** is the number of base; **TotalReads** is the number of reads; **MaxLen** is maximum length of data; **AvgLen** is average length of data; **N50** is N50 of data, all reads are summed in order from long to short, and when additive length up to half of all reads total length, the last read length added is N50; **L50** is L50 of data, all reads are accumulated in turn ranking in order from long to short, when additive length up to half of all reads total length, the number of sequences is L50; **N90** is N90 of data, algorithm the same as N50; **L90** is L90 of data, algorithm the same asL50; **meanQ** is mean quality value.

Table S3. Statistics of Illumina NovaSeq sequencing data volume information of *I. hispidus* NPCB_001 genome.

Sample_name	Total_reads	Total_bases	GC_content	Q20	Q30
rawdata	38,199,760	5,729,964,000	47.76%	97.85%	93.72%
cleandata	38,199,760	5,729,964,000	47.76%	97.85%	93.72%

Sample name is data type; **Total_reads** is sequencing reads number; **Total_bases** is total sequencing base number; **GC_Content** is G/C base number as a percentage of total base number; **Q20**、**Q30** respectively are Phred value greater than 20、30 base as a percentage of total base.

Table S4. Statistics of Illumina NovaSeq sequencing data mapping of *I. hispidus* NPCB_001 genome.

Type	Number
map_rate	97.91%
Average_depth	163.36
Coverage	99.86%

map_rate is Illumina NovaSeq sequencing data mapping rates; **Average_depth** is average coverage depth; **Coverage** is coverage rate.

Table S5. Annotation Statistics of *I. hispidus* NPCB_001 coding gene.

Type	Number
the total number of gene	12,304
the average of mRNA_length	1,816.15
the average of cds_length	1,434.49
the average of exon_number	6.40
the average of exon_length	224.07
the average of intron_length	70.65
the total number of exon	78,769
the total number of intron	66,465
the total intron length	4,695,439

the total number of gene is the total number of genes; **the average of mRNA_length** is the average length of mRNA; **the average of cds_length** is the average length of CDS; **the average of exon_number** is the average number of exons per gene. **the average of exon_length** is the average exon length; **the average of intron_length** is the average intron length; **the total number of exon** is the total number of exons; **the total number of intron** is the total number of introns; **the total intron length** is the total intron length.

Table S6. Statistics of non-coding RNA annotation results in *I. hispidus* NPCB_ 001 genome.

Class	number	totalLen(bp)	meanLen(bp)
rRNA	14	25,246	1,803
sRNA	1	344	344
snRNA	16	1,962	122
tRNA	105	8,353	79

rRNA is ribosomal RNA; tRNA is transport RNA; sRNA is small regulatory RNA; snRNA is nucleolar small RNA. **totalLen** and **meanLen** are the total length and mean length.

Table S7. Statistics of *I. hispidus* NPCB_001 repetitive sequence annotation results.

Item	Subfamily	Number	Length(bp)	Coverage
SINE	/	2	162	0.00%
LINE	/	233	20,701	0.06%
LTR	/	1,384	747,007	2.20%
LTR	Gypsy	1,076	656,064	1.93%
LTR	Copia	226	85,202	0.25%
DNA	/	407	77,360	0.23%
Satellite	/	7	486	0.00%
Simple_repeat	/	5,023	201,468	0.59%
Low_complexity	/	857	40,901	0.12%
Other	/	40	8,243	0.02%
Unknown	/	34	7,413	0.02%
Total	/	7,987	1,100,314	3.23%

SINE is short scattered element; **LINE** is Long scattered element; **LTR** is long terminal repetition, mainly include two types, Gypsy and Copia; **DNA** is transposons; **Satellite** is satellite repetitive sequence; **Low_complexity** is Low_complexity repetition; **Other** is other types repetition; **Unknown** is unknown repetitive sequence; **Total** is total repetitive sequence.

Table S8. Statistics of *I. hispidus* NPCB_001 protein-coding gene annotation.

Item	Count	Percentage
Uniprot	5,760	46.81%
Pfam	8,401	68.28%
Refseq	4,295	34.91%
Nr	10,580	85.99%
Interproscan	8,401	68.28%
GO	5,719	46.48%
KEGG	3,900	31.70%
Pathway	2,375	19.30%
COG	1,051	8.54%
Annotation	10,814	87.89%
All	12,304	100%

Annotation is the gene with at least one annotation; Uniprot is the gene annotated to the Uniprot database; Pfam is the gene that is annotated to the Pfam database; Refseq is the gene that is annotated to the Refseq database; Nr is the gene that is annotated to the Nr database; Interproscan is the gene that is annotated to the Interproscan GO is the gene annotated to the GO database; KEGG is the gene that is annotated to the KEGG database; Pathway is the gene that is annotated to the KEGG Pathway database; COG is the gene that is annotated to the COG database.

Table S9. Candidate genes for mating type in I. hispidus NPCB_001 genome.

**Top Annotation**	**Gene ID**	**Species**	**E -Value**	**Accession Number**
mitochondrial intermediate peptidase (MIP)	g1048	*Schizosaccharomyces pombe*	$3e^{-52}$	Q9Y7U6.1
unknown conserved fungal protein-codon gene (*βFG*)	g9997	*Fomitiporia mediterranea*	$2e^{-70}$	EJD03593.1
homeodomain protein	g1045	*Kluyveromyces lactis*	$1e^{-56}$	P07921.1
	g1047	*Sanghuangporus baumii*	$3e^{-103}$	OCB84704.1
HD1 mating type protein	g1046	*Fomitiporia mediterranea*	$9e^{-48}$	EJD06593.1
pheromone receptors (*ste3*)	g3868	*Schizophyllum commune*	$6e^{-61}$	P56502.2
	g3904	*Schizophyllum commune*	$8e^{-53}$	P56502.2
	g4149	*Schizophyllum commune*	$2e^{-95}$	P56502.2
glycosyltransferase family 8 protein	g9996	*Rattus norvegicus*	$4e^{-41}$	O08730.4

Table S10. The source (URL) statistics for 47 representative Basidiomycetes used to phylogenetic analysis.

Species	Source
Malassezia sympodialis	https://www.ncbi.nlm.nih.gov/genome/?term=Malassezia%20sympodialis
Tilletiaria anomala	https://www.ncbi.nlm.nih.gov/genome/?term=Tilletiaria%20anomala
Ustilago maydis	https://www.ncbi.nlm.nih.gov/genome/?term=Ustilago%20maydis
Puccinia striiformis	https://www.ncbi.nlm.nih.gov/genome/?term=Puccinia%20striiformis
Rhodotorula toruloides	https://www.ncbi.nlm.nih.gov/genome/?term=Rhodotorula%20toruloides
Wallemia mellicola	https://www.ncbi.nlm.nih.gov/genome/?term=Wallemia%20mellicola
Filobasidium floriforme	https://www.ncbi.nlm.nih.gov/genome/?term=Filobasidium%20floriforme
Cryptococcus depauperatus	https://www.ncbi.nlm.nih.gov/genome/?term=Cryptococcus%20depauperatus
Trichosporon asahii	https://www.ncbi.nlm.nih.gov/genome/?term=Trichosporon%20asahii
Calocera cornea	https://www.ncbi.nlm.nih.gov/genome/?term=Calocera%20cornea
Hydnum rufescens	https://www.ncbi.nlm.nih.gov/genome/?term=Hydnum%20rufescens
Serendipita vermifera	https://www.ncbi.nlm.nih.gov/genome/?term=Serendipita%20vermifera
Auricularia subglabra	https://www.ncbi.nlm.nih.gov/genome/?term=Auricularia%20subglabra
Gloeophyllum trabeum	https://www.ncbi.nlm.nih.gov/genome/?term=Gloeophyllum%20trabeum
Stereum hirsutum	https://www.ncbi.nlm.nih.gov/genome/?term=Stereum%20hirsutum
Hericium alpestre	https://www.ncbi.nlm.nih.gov/genome/?term=Hericium%20alpestre
Cristinia sonorae	https://www.ncbi.nlm.nih.gov/genome/?term=Cristinia%20sonorae
Ganoderma sinense	https://www.ncbi.nlm.nih.gov/genome/?term=Ganoderma%20sinense
Gelatoporia subvermispora	https://www.ncbi.nlm.nih.gov/genome/?term=Gelatoporia%20subvermispora
Wolfiporia cocos	https://www.ncbi.nlm.nih.gov/genome/?term=Wolfiporia%20cocos
Laetiporus sulphureus	https://www.ncbi.nlm.nih.gov/genome/?term=Laetiporus%20sulphureus
Suillus brevipes	https://www.ncbi.nlm.nih.gov/genome/?term=Suillus%20brevipes
Schizophyllum commune	https://www.ncbi.nlm.nih.gov/genome/?term=Schizophyllum%20commune
Pteruala gracilis	https://www.ncbi.nlm.nih.gov/genome/?term=pterula%20gracilis

Pleurotus ostreatus	https://www.ncbi.nlm.nih.gov/genome/?term=Pleurotus%20ostreatus
Marasmius oreades	https://www.ncbi.nlm.nih.gov/genome/?term=Marasmius%20oreades
Dendrothele bispora	https://www.ncbi.nlm.nih.gov/genome/?term=Dendrothele%20bispora
Lentinula edodes	https://www.ncbi.nlm.nih.gov/genome/?term=Lentinula%20edodes
Gymnopus androsaceus	https://www.ncbi.nlm.nih.gov/genome/?term=Gymnopus%20androsaceus
Amanita muscaria	https://www.ncbi.nlm.nih.gov/genome/?term=Amanita%20muscaria
Pluteus cervinus	https://www.ncbi.nlm.nih.gov/genome/?term=Pluteus%20cervinus
Tricholoma matsutake	https://www.ncbi.nlm.nih.gov/genome/?term=Tricholoma%20matsutake
Lyophyllum atratum	https://www.ncbi.nlm.nih.gov/genome/?term=Lyophyllum%20atratum
Agaricus bisporus	https://www.ncbi.nlm.nih.gov/genome/?term=Agaricus%20bisporus
Psilocybe cubensis	https://www.ncbi.nlm.nih.gov/genome/?term=Psilocybe%20cubensis
Cortinarius glaucopus	https://www.ncbi.nlm.nih.gov/genome/?term=Cortinarius%20glaucopus
Crassisporium funariophilum	https://www.ncbi.nlm.nih.gov/genome/?term=Crassisporium%20funariophilum
Rickenella mellea	https://www.ncbi.nlm.nih.gov/genome/?term=Rickenella%20mellea
Schizopora paradoxa	https://www.ncbi.nlm.nih.gov/genome/?term=Schizopora%20paradoxa
Phellinidium pouzari	https://www.ncbi.nlm.nih.gov/genome/?term=Phellinidium%20pouzarii
Phellopilus nigrolimitatus	https://www.ncbi.nlm.nih.gov/genome/?term=Phellopilus%20nigrolimitatus
Pyrrhoderma noxium	https://www.ncbi.nlm.nih.gov/genome/?term=Pyrrhoderma%20noxium
Phellinus noxius	https://www.ncbi.nlm.nih.gov/genome/?term=Phellinus%20noxius
Porodaedalea chrysoloma	https://genome.jgi.doe.gov/portal/pages/dynamicOrganismDownload.jsf?organism=Porchr1
Sanghuangporus baumii	https://www.ncbi.nlm.nih.gov/genome/?term=Sanghuangporus%20baumii
Inonotus obliquus	https://www.ncbi.nlm.nih.gov/genome/?term=Inonotus%20obliquus
Inonotus hispidus	https://www.ncbi.nlm.nih.gov/genome/?term=Inonotus%20hispidus

Table S11. The source (URL) statistics for white-rot fungi used to analysis CAZymes.

Species	Source
Inonotus hispidus NPCB_001	the study
Armillaria ostoyae C18/9	https://www.ncbi.nlm.nih.gov/genome/?term=Armillaria+ostoyae
Auricularia subglabra SS-5 V1.0	https://www.ncbi.nlm.nih.gov/genome/?term=Auricularia+subglabra
Cerioporus squamosus CCBS 676	https://www.ncbi.nlm.nih.gov/genome/?term=Cerioporus+squamosus
Ceriporiopsis subvermispora B v1.0	https://www.ncbi.nlm.nih.gov/genome/?term=Cerioporus%20squamosus
Dichomitus squalens LYAD-421 SS1	https://www.ncbi.nlm.nih.gov/genome/?term=Dichomitus%20squalens
Fomes fomentarius CIRM-BRFM 1821	https://www.ncbi.nlm.nih.gov/genome/?term=Fomes+fomentarius
Ganoderma boninense G3 v1.0	https://genome.jgi.doe.gov/portal/pages/dynamicOrganismDownload.jsf?organism=Ganbon1
Ganoderma leucocontextum Dai12418 v1.0	https://genome.jgi.doe.gov/portal/pages/dynamicOrganismDownload.jsf?organism=Ganleu1
Ganoderma lucidum G.260125-1 v1.0	https://genome.jgi.doe.gov/portal/pages/dynamicOrganismDownload.jsf?organism=Ganluc1
Ganoderma sinense ZZ0214-1	https://www.ncbi.nlm.nih.gov/genome/?term=Ganoderma%20sinense
Heterobasidion annosum v2.0	https://genome.jgi.doe.gov/portal/pages/dynamicOrganismDownload.jsf?organism=Hetan2
Irpex lacteus CCBAS Fr. 238 617/93 v1.0	https://genome.jgi.doe.gov/portal/pages/dynamicOrganismDownload.jsf?organism=Irplac1
Irpex rosettiformis CBS 384.51	https://www.ncbi.nlm.nih.gov/genome/?term=Irpex+rosettiformis
Lentinula edodes Le (Bin) 0899 ss11	https://www.ncbi.nlm.nih.gov/genome/?term=Lentinula+edodes
Mucidula mucida CBS 558.79	https://www.ncbi.nlm.nih.gov/genome/?term=Mucidula%20mucida
Panus rudis PR-1116 ss-1	https://www.ncbi.nlm.nih.gov/genome/?term=Panus+rudis
Penicillium chrysogenum ASM71027v1	https://www.ncbi.nlm.nih.gov/genome/?term=Penicillium+chrysogenum
Penicillium decumbens ASM207224v1	https://www.ncbi.nlm.nih.gov/genome/?term=Penicillium+decumbens
Penicillium subrubescens ASM190812v1	https://www.ncbi.nlm.nih.gov/genome/?term=Penicillium+subrubescens
Phanerochaete carnosa HHB-10118-Sp v1.0	https://genome.jgi.doe.gov/portal/pages/dynamicOrganismDownload.jsf?organism=Phaca1
Phanerochaete chrysosporium RP-78 v2.2	https://genome.jgi.doe.gov/portal/pages/dynamicOrganismDownload.jsf?organism=Phchr2
Phlebia brevispora HHB-7030 SS6 v1.0	https://genome.jgi.doe.gov/portal/pages/dynamicOrganismDownload.jsf?organism=Phlbr1
Phlebia centrifuga FBCC195	https://genome.jgi.doe.gov/portal/pages/dynamicOrganismDownload.jsf?organism=Phlcen1

Pleurotus cornucopiae ASM1967732v2	https://www.ncbi.nlm.nih.gov/genome/?term=Pleurotus+cornucopiae
Pleurotus ostreatoroseus ASM529804v1	https://www.ncbi.nlm.nih.gov/genome/?term=Pleurotus%20ostreatoroseus
Pleurotus ostreatus PC9	https://www.ncbi.nlm.nih.gov/genome/?term=Pleurotus+ostreatus
Polyporus arcularius HHB13444	https://www.ncbi.nlm.nih.gov/genome/?term=Polyporus+arcularius
Polyporus squamosus CCBS 676 v1.0	https://genome.jgi.doe.gov/portal/pages/dynamicOrganismDownload.jsf?organism=Polsqu1
Stereum hirsutum FP-91666 SS1	https://www.ncbi.nlm.nih.gov/genome/?term=Stereum+hirsutum
Trametes cingulata BRFM 1805	https://www.ncbi.nlm.nih.gov/genome/?term=Trametes+cingulata
Trametes gibbosa CIRM-BRFM 1770 v1.0	https://genome.jgi.doe.gov/portal/pages/dynamicOrganismDownload.jsf?organism=Tragib1
Trametes maxima CIRM-BRFM 1813 v1.0	https://genome.jgi.doe.gov/portal/pages/dynamicOrganismDownload.jsf?organism=Tramax1
Trametes polyzona CIRM-BRFM 1798 v1.0	https://genome.jgi.doe.gov/portal/pages/dynamicOrganismDownload.jsf?organism=Trapol1
Trametes versicolor FP-101664 SS1	https://www.ncbi.nlm.nih.gov/genome/?term=Trametes+versicolor
Trichaptum abietinum v1.0	https://genome.jgi.doe.gov/portal/pages/dynamicOrganismDownload.jsf?organism=Triab1_1
Trichoderma asperellum CBS 433.97	https://www.ncbi.nlm.nih.gov/genome/?term=Trichoderma+asperellum
Trichoderma harzianum CBS 226.95	https://www.ncbi.nlm.nih.gov/genome/?term=Trichoderma+harzianum
Trichoderma reesei QM6a v2.0	https://genome.jgi.doe.gov/portal/pages/dynamicOrganismDownload.jsf?organism=Trire_Chr

Table S12. The 31 candidate genes for polysaccharide biosynthesis screened in the database.

Gene ID	Type	Uniprot	Pfam	Refseq	Nr	Interproscan	COG	KEGG	Pathway	GO
g10984.t1	GK	√	√	√		√	√	√	√	√
g1643.t1	GK	√		√	√	√	√	√	√	√
g9496.t1	PGM	√	√	√	√	√	√	√	√	√
g1845.t1	PGM		√	√	√	√		√	√	√
g6624.t1	PGM	√	√				√			√
g5619.t1	PGM						√	√		
g11146.t1	PGM	√					√			√
g7966.t1	PGM	√	√	√	√	√		√	√	√
g1992.t1	PGM			√	√	√		√	√	
g2104.t1	GLS	√	√		√	√		√	√	√
g407.t1	GLS	√	√		√	√		√	√	√
g6073.t1	GSAP		√		√	√				√
g9853.t1	GSAP		√	√	√	√				√
g11429.t1	GSAP	√	√	√	√	√				√
g11374.t1	GSAP	√	√	√	√	√				√
g9723.t1	GSAP		√	√		√				√
g6104.t1	PMI	√	√	√	√	√		√	√	√
g958.t1	PMI	√	√	√	√	√	√	√	√	√
g1749.t1	PGI	√	√	√	√	√		√	√	√
g2234.t1	FBPase	√	√	√	√	√		√	√	√
g944.t1	UGE	√	√							√
g10160.t1	UGE	√	√	√	√	√	√	√	√	√
g10636.t1	UXS	√					√	√	√	√
g6344.t1	GMD		√					√		
g684.t1	GMD	√	√	√		√	√	√	√	√
g8023.t1	GMD		√				√			√
g714.t1	GMD	√	√	√	√	√		√	√	√
g8855.t1	GMD		√							
g1379.t1	GMD		√							
g2664.t1	GMD		√			√	√	√		
g6775.t1	FPK	√	√	√	√	√		√	√	√

Table S13. The candidate genes for polysaccharide biosynthesis in *I. hispidus* NPCB_001 genome.

Top Annotation	Gene ID	Species	E - Value	Per.Ident	Accession Number
Glucokinase (GK)	g10984.t1	*Aspergillus niger*	$4e^{-115}$	41.98%	Q92407.1
	g1643.t1	*Gluconobacter oxydans*	$3e^{-30}$	41.34%	Q5FQ97.1
Phosphoglucomutase/ Glucose phosphomutase (PGM)	g9496.t1	*Xanthobacter autotrophicus*	$1e^{-11}$	26.68%	A7IIG5.2
	g1845.t1	*Pongo abelii*	$7e^{-163}$	43.99%	Q5R979.1
	g6624.t1	*Saccharomyces cerevisiae*	$1e^{-11}$	28.76%	P38773.1
	g5619.t1	*Bacillus subtilis*	$6e^{-5}$	25.45%	O06995.1
	g11146.t1	*Bacillus subtilis*	$4e\text{-}^{5}$	23.32%	O06995.1
	g7966.t1	*Aspergillus fumigatus*	0	60.39%	Q4WY53.1
	g1992.t1	*Haloferax volcanii*	$3e^{-4}$	25.19%	D4GYH1.1
1,3-beta-glucan synthase (GLS)	g2104.t1	*Cryptococcus neoformans*	0	71.83%	O93927.3
	g407.t1	*Cryptococcus neoformans*	0	73.71%	O93927.3
Beta-glucan synthesis-associated protein (GSAP)	g6073.t1	*Saccharomyces cerevisiae*	$3e^{-116}$	45.37%	P32486.2
	g9853.t1	*Candida albicans*	$1e^{-112}$	39.93%	P87024.1
	g11429.t1	*Saccharomyces cerevisiae*	$1e^{-117}$	45.85%	P33336.1
	g11374.t1	*Saccharomyces cerevisiae*	$7e^{-109}$	44.95%	P33336.1
	g9723.t1	*Trichophyton benhamiae*	$5e^{-52}$	38.41%	D4AJR9.1
Phosphomannose isomerase/Mannose-6-phosphate isomerase (PMI)	g6104.t1	*Candida albicans*	$8e^{-86}$	35.19%	P34948.2
	g958.t1	*Cryptococcus neoformans*	$2e^{-117}$	45.47%	Q9HFU4.2
Glucose-6-phosphate isomerase / Phosphoglucose isomerase (PGI)	g1749.t1	*Agaricus bisporus*	0	77.49%	Q711G1.1
Fructose-1,6-bisphosphatase (FBPase)	g2234.t1	*Spinacia oleracea*	$7e^{-135}$	61.33%	P14766.2
UDP-glucose 4-epimerase (UGE)	g944.t1	*Oryza sativa*	$3e^{-8}$	28.92%	Q6K2E1.1
	g10160.t1	*Dictyostelium discoideum*	$2e^{-85}$	45.01%	Q553X7.1

UDP-glucuronic acid decarboxylase 1 (UXS)	g10636.t1	*Mus musculus*	$5e^{-154}$	57.42%	Q91XL3.1
GDP-mannose 4,6 dehydratase (GMD)	g6344.t1	*Azospirillum brasilense*	$1e^{-11}$	26.27%	Q59083.1
	g684.t1	*Mus musculus*	$3e^{-124}$	55.36%	P23591.3
	g8023.t1	*Mycoplasma genitalium*	0.012	27.66%	P47364.1
	g714.t1	*Cricetulus griseus*	$1e^{-178}$	69.36%	Q8K3X3.1
	g8855.t1	*Rickettsia bellii*	0.035	30.38%	Q1RIM4.1
	g1379.t1	*Rickettsia bellii*	0.007	30.77%	Q1RIM4.1
	g2664.t1	*Mycobacterium tuberculosis*	$3e^{-12}$	36.04%	P9WH08.1
Phosphofructokinase (FPK)	g6775.t1	*Schizosaccharomyces pombe*	0	53.87%	O42938.1

Table S14. Terpenoid biosynthesis related enzymes in *I. hispidus* NPCB_001.

gene ID	protein ID	Top Annotation				Type
		Identities	**E -Value**	**Species**	**Accession Number**	
g7579	g7579.t1	65.88%	0	*Ganoderma lucidum*	D7NJ68.1	oxidosqualene cyclase
g8354	g8354.t1	60.94%	0	*Ganoderma lucidum*	A0SJQ5.1	squalene synthase
g7183	g7183.t1	57.41%	$3e^{-136}$	*Cyclocybe aegerita*	A0A5Q0QRJ3.1	sesquiterpene synthase
g8436	g8436.t1	53.45%	$1e^{-145}$	*Postia placenta*	A0A348B788.1	
g8982	g8982.t1	32.51%	$1e^{-52}$	*Postia placenta*	B8PD44.1	
g8985	g8985.t1	35.08%	$7e^{-59}$	*Postia placenta*	B8PD44.1	
g8990	g8990.t1	27.16%	$7e^{-30}$	*Postia placenta*	B8PD44.1	
g8991	g8991.t1	31.91%	$1e^{-51}$	*Postia placenta*	B8PD44.1	
g8992	g8992.t1	28.47%	$5e^{-31}$	*Postia placenta*	B8PD44.1	
g9225	g9225.t1	30.82%	$3e^{-47}$	*Postia placenta*	B8PD44.1	
g9226	g9226.t1	28.99%	$1e^{-38}$	*Postia placenta*	B8PD44.1	
g9227	g9227.t1	31.99%	$8e^{-42}$	*Postia placenta*	B8PD44.1	
g1390	g1390.t1	66.08%	$6e^{-172}$	*Coprinopsis cinerea*	A8NE23.1	
g6332	g6332.t1	59.65%	$8e^{-155}$	*Postia placenta*	A0A348B788.1	
g2983	g2983.t1	57.41%	$3e^{-136}$	*Cyclocybe aegerita*	A0A5Q0QRJ3.1	
g1139	g1139.t1	45.71%	$2e^{-100}$	*Postia placenta*	B8PD44.1	
g8866	g8866.t1	66.08%	$6e^{-172}$	*Coprinopsis cinerea*	A8NE23.1	
g6286	g6286.t1	37.13%	$5e^{-56}$	*Rattus norvegicus*	D3ZN43.1	phytoene synthase

Table S15. Identify matrix of Eight STSs from *I. hispidus* NPCB_001.

	g9226.t1	g8991.t1	g9227.t1	g8985.t1	g8982.t1	g8992.t1	g9225.t1	g8990.t1
g9226.t1	100	69.83	43.3	46.44	49.49	42.69	43.05	42.97
g8991.t1	69.83	100	46.37	49.7	50.6	44.56	45.54	45.07
g9227.t1	43.3	46.37	100	90.91	51.42	41.58	43.85	43.16
g8985.t1	46.44	49.7	90.91	100	55.12	43.45	45.78	44.67
g8982.t1	49.49	50.6	51.42	55.12	100	54.42	55.36	53.62
g8992.t1	42.69	44.56	41.58	43.45	54.42	100	91.19	93.16
g9225.t1	43.05	45.54	43.85	45.78	55.36	91.19	100	90.82
g8990.t1	42.97	45.07	43.16	44.67	53.62	93.16	90.82	100

The percent identity matrix was analyzed online by Clustal Omega (https://www.ebi.ac.uk/Tools/msa/clustalo/).

Table S16. NRPS-like biosynthesis related enzymes in *I. hispidus* NPCB_001 genome.

gene ID	protein ID	Top Annotation					Type
		homology	Identities	**E -Value**	**Species**	**Accession Number**	
g7927	g7927.t1	Nps10	28.07%	$3e^{-88}$	*Heterobasidion annosum*	A0A1B1ZGB5.1	NRPS-like
g8202	g8202.t1	Nps10	38.35%	0	*Heterobasidion annosum*	A0A1B1ZGB5.1	NRPS-like
g8310	g8310.t1	HispS	26.50%	$1e^{-100}$	*Neonothopanus nambi*	A0A3G9K3K9.1	PKS-NRPS hybrid
g8482	g8482.t1	greA	35.64%	$7e^{-177}$	*Suillus grevillei*	I6NXV7.1	NRPS-like
g1509	g1509.t1	Lys1	48.94%	0	*Schizosaccharo myces pombe*	P40976.3	L-2-aminoadipa te reductase

Table S17. The 46 identified NRPS-likes from fungi used in cluster analysis.

Entry	Genbank Accession No	Fungal species
ATEG_03630	Q0CRQ4	*Aspergillus terreus*
StbB	A0A193PS46	*Stachybotrys bisbyi*
CicB	A0A1U8QW91	*Emericella nidulans*
Nps11	F8P9P5	*Serpula lacrymans var. lacrymans*
Nps9	F8P2C8	*Serpula lacrymans var. lacrymans*
NCU05000	Q7RW48	*Neurospora crassa*
Nps10	A0A1B1ZGB5	*Heterobasidion annosum*
ATRR	A0A1U8QWA2	*Emericella nidulans*
Nps3	F8P1W3	*Serpula lacrymans var. lacrymans*
InvA2	A0A0S1RUN4	*Paxillus involutus*
InvA1	A0A0S2E7Z1	*Paxillus involutus*
InvA5	A0A0S2E7W7	*Paxillus involutus*
GreA	I6NXV7	*Suillus grevillei*
CC1G_06235	A8NVB7	*Coprinopsis cinerea*
CC1G_03009	A8NS27	*Coprinopsis cinerea*
InvA3	A0A0S2E7V8	*Paxillus involutus*
ATEG_03629	Q0CRQ5	*Aspergillus terreus*
InvA4	A0A0S2E7X0	*Paxillus involutus*
InvA6	A0A0S2E7W3	*Paxillus involutus*
ATEG_03631	Q0CRQ3	*Aspergillus terreus*
ATEG_07661	Q0CF73	*Aspergillus terreus*
MelA	Q0CRX1	*Aspergillus terreus*
ATEG_07662	Q0CF72	*Aspergillus terreus*
LnaA	B8NTZ9	*Aspergillus flavus*
ATEG_07659	Q0CF75	*Aspergillus terreus*
ApvA	Q0CWD0	*Aspergillus terreus*
MicA	Q5B7T4	*Emericella nidulans*
LnbA	B8NWW5	*Aspergillus flavus*
StbA	A0A193PS74	*Stachybotrys bisbyi*
LnaD	B8NU02	*Aspergillus flavus*
ATEG_03635	Q0CRP9	*Aspergillus terreus*
LnaC	B8NU01	*Aspergillus flavus*
ATEG_07660	Q0CF74	*Aspergillus terreus*
LnbC	B8NWW3	*Aspergillus flavus*
BtyA	Q0CU19	*Aspergillus terreus*
StbC	A0A193PS58	*Stachybotrys bisbyi*
PngA	Q0CBN5	*Aspergillus terreus*
AtrA	Q0CT94	*Aspergillus terreus*
ATEG_07663	Q0CF71	*Aspergillus terreus*
LnaF1	B8NU03	*Aspergillus flavus*
LnaB1	B8NU00	*Aspergillus flavus*
ATEG_03636	Q0CRP8	*Aspergillus terreus*
LnaE	B8NTZ8	*Aspergillus flavus*
LnaB2	B8NWW2	*Aspergillus flavus*
LnaF2	B8NWW7	*Aspergillus flavus*
LnbE	B8NWW6	*Aspergillus flavus*

Table S18. Sequence homology analysis for two PKSs from *I. hispidus* NPCB_001.

gene ID	protein ID	Top Annotation				
		homologous	Identities	**E -Value**	**Species**	**Accession Number**
g1510	g1510.t1	PikA2	33.60%	1e^{-62}	*Streptomyces venezuelae*	Q9ZGI4.1
g6596	g6596.t1	ArmB	40.24%	0	*Armillaria ostoyae*	A0A284RE13.1

Table S19. The 22 identified PKS from Basidiomycetes used in cluster analysis.

Uniport Entry	Gene Names	Protein	Annotations	Organism
A0A0D1DNX1	fas2, UMAG_06460	Fas2	Fatty acid synthase 2	*Ustilago maydis*
O43103	SID2, UMAG_10189	Sid2	Ferrichrome siderophore peptide synthetase	*Ustilago maydis*
Q4PEM9	fer3, UMAG_01434	Fer3	Siderophore peptide synthetase fer3	*Ustilago maydis*
B7STY1	atrA	AtrA	Atromentin synthetase	*Tapinella panuoides*
I6NXV7	greA	GreA	Atromentin synthetase greA	*Suillus grevillei*
A0A384XH94	stpks1	StPKS1	Highly reducing polyketide synthase stpks1	*Strobilurus tenacellus*
F8P1W3	nps3, SERLADRAFT_416588	Nps3	Atromentin synthetase nps3	*Serpula lacrymans var. lacrymans*
F8P9P5	nps11, SERLADRAFT_453044	Nps11	Adenylate-forming reductase Nps11	*Serpula lacrymans var. lacrymans*
A0A0S1RUN4	invA2	InvA2	i	*Paxillus involutus*
A0A0S2E7V8	invA3	InvA3	Inactive atromentin synthetase invA3	*Paxillus involutus*
A0A0S2E7W3	invA6	InvA6	Inactive atromentin synthetase invA6	*Paxillus involutus*
A0A0S2E7W7	invA5	InvA5	Atromentin synthetase invA5	*Paxillus involutus*
A0A0S2E7X0	invA4	InvA4	Inactive atromentin synthetase invA4	*Paxillus involutus*
A0A0S2E7Z1	invA1	InvA1	Atromentin synthetase invA1	*Paxillus involutus*
Q52US9	fso1	Fso1	Nonribosomal peptide synthase fso1	*Omphalotus olearius*
A0A3G9K3K9	hisps	HispS	Hispidin synthase	*Neonothopanus nambi*
A0A1B1ZGB5	nps10	Nps10	Adenylate-forming reductase Nps10	*Heterobasidion annosum*
A8NF97	cpf1, CC1G_04210	Cpf1	Coprinoferrin synthetase	*Coprinopsis cinerea*
A0A248AFK6	NPS2, CERSUDRAFT_172109	NPS2	Nonribosomal peptide synthase NPS2	*Ceriporiopsis subvermispora*
A0A284RE13	armB, ARMOST_10343	AO_ArmB	Orsellinic acid synthase ArmB	*Armillaria ostoyae*
I3ZNU9	armB	AM_ArmB	Orsellinic acid synthase armB	*Armillaria mellea*
A0A2H3CTK0	armB, ARMGADRAFT_1169973	AG_ArmB	Orsellinic acid synthase armB	*Armillaria gallica*

Table S20. Identification of cytochrome P450 genes in *I. hispidus* NPCB_001 genome.

Protein ID	Gene ID	aa length	Identify	E-Value	Species	Accession
g7737.t1	g7737	555	42.63%	$8e^{-119}$	*Postia placenta*	F1SYD1.1
g7738.t1	g7738	285	37.34%	$6e^{-51}$	*Postia placenta*	F1SY95.1
g7739.t1	g7739	215	56.59%	$7e^{-81}$	*Postia placenta*	F1SYD1.1
g7740.t1	g7740	577	41.84%	$2e^{-136}$	*Postia placenta*	F1SYD1.1
g7892.t1	g7892	551	32.11%	$3e^{-72}$	*Postia placenta*	F1SYB2.1
g5143.t1	g5143	547	33.33%	$9e^{-53}$	*Postia placenta*	F1SYB2.1
g5144.t1	g5144	546	32.10%	$5e^{-55}$	*Postia placenta*	F1SYB2.1
g6498.t1	g6498	511	25.15%	$1e^{-40}$	*Postia placenta*	F1SYB2.1
g9922.t1	g9922	540	49.79%	$3e^{-142}$	*Postia placenta*	F1SYB2.1
g2535.t1	g2535	523	54.74%	0	*Postia placenta*	F1SYB2.1
g2443.t1	g2443	556	27.55%	$4e^{-50}$	*Postia placenta*	F1SYB2.1
g328.t1	g328	576	29.10%	$1e^{-53}$	*Postia placenta*	F1SYB2.1
g8153.t1	g8153	496	27.84%	$1e^{-56}$	*Postia placenta*	F1SYH0.1
g11557.t1	g11557	538	34.58%	$4e^{-87}$	*Postia placenta*	F1SYE5.1
g11568.t1	g11568	531	32.97%	$1e^{-92}$	*Postia placenta*	F1SYE5.1
g9242.t1	g9242	376	32.25%	$1e^{-37}$	*Postia placenta*	F1SYE5.1
g5325.t1	g5325	560	36.45%	$6e^{-99}$	*Postia placenta*	F1SYH0.1
g9243.t1	g9243	184	43.26%	$4e^{-44}$	*Postia placenta*	F1SYH7.1
g5945.t1	g5945	616	37.34%	$2e^{-105}$	*Postia placenta*	F1SYH7.1
g808.t1	g808	548	52.38%	0	*Postia placenta*	F1SYE5.1
g1614.t1	g1614	547	49.13%	$3e^{-176}$	*Postia placenta*	F1SY85.1
g5827.t1	g5827	547	44.42%	$1e^{-166}$	*Postia placenta*	F1SY85.1
g5903.t1	g5903	473	48.42%	$4e^{-163}$	*Postia placenta*	F1SY85.1
g5813.t1	g5813	444	49.41%	$5e^{-150}$	*Postia placenta*	F1SY85.1
g5828.t1		545	46.68%	$3e^{-178}$	*Postia placenta*	F1SY85.1
g5828.t3	g5828	526	43.36%	$5e^{-155}$	*Postia placenta*	F1SY85.1
g5828.t2		496	47.74%	$3e^{-166}$	*Postia placenta*	F1SY85.1
g6234.t1	g6234	606	47.16%	$5e^{-156}$	*Postia placenta*	F1SY85.1
g5881.t1	g5881	543	48.28%	$7e^{-172}$	*Postia placenta*	F1SYH4.1
g5882.t1	g5882	553	47.07%	$3e^{-172}$	*Postia placenta*	F1SYI1.1
g5885.t1	g5885	543	47.78%	$8e^{-179}$	*Postia placenta*	F1SYH4.1
g3909.t1	g3909	411	40.71%	$8e^{-98}$	*Postia placenta*	F1SY85.1
g2430.t1	g2430	613	36.52%	$2e^{-115}$	*Postia placenta*	F1SYH4.1
g3331.t1	g3331	546	34.05%	$4e^{-68}$	*Postia placenta*	F1SYB6.1
g8227.t1	g8227	510	28.30%	$4e^{-41}$	*Mus musculus*	Q9WVK8.1
g10492.t1	g10492	591	31.03%	$3e^{-69}$	*Penicillium brefeldianum*	A0A068AA78.1
g1801.t1	g1801	574	30.14%	$1e^{-56}$	*Penicillium brefeldianum*	A0A068AA78.1

g10583.t1	g10583	585	30.61%	$4e^{-67}$	*Uromyces viciae-fabae*	O00061.1
g1482.t1	g1482	576	33.66%	$2e^{-64}$	*Uromyces viciae-fabae*	O00061.1
g1791.t1	g1791	574	32.74%	$7e^{-70}$	*Uromyces viciae-fabae*	O00061.1
g1802.t1	g1802	648	31.83%	$2e^{-59}$	*Fusarium pseudograminearum*	P0DPA4.1
g8590.t1	g8590	598	32.72%	$1e^{-74}$	*Fusarium pseudograminearum*	P0DPA4.1
g8598.t1	g8598	564	31.91%	$1e^{-74}$	*Fusarium pseudograminearum*	P0DPA4.1
g2592.t1	g2592	543	45.09%	$3e^{-162}$	*Postia placenta*	F1SY83.1
g2721.t1	g2721	516	45.16%	$1e^{-144}$	*Postia placenta*	F1SY49.1
g344.t1	g344	576	25.05%	$2e^{-30}$	*Fusarium graminearum*	I1RV19.1
g8167.t1	g8167	412	27.86%	$5e^{-45}$	*Ustilago maydis*	E9RHV4.1
g10139.t1	g10139	581	48.86%	$8e^{-167}$	*Postia placenta*	F1SY74.1
g10141.t1	g10141	551	48.25%	0	*Postia placenta*	F1SY74.1
g10142.t1	g10142	613	50.98%	0	*Postia placenta*	F1SY74.1
g4881.t1	g4881	885	39.77%	$2e^{-139}$	*Postia placenta*	F1SY74.1
g10551.t1	g10551	581	53.05%	0	*Ustilago maydis*	P49602.1
g894.t1	g894	553	58.85%	0	*Ustilago maydis*	P49602.1
g11566.t1	g11566	493	29.31%	$4e^{-44}$	*Aspergillus violaceofuscus*	A0A2V5GZ95.1
g11567.t1	g11567	1049	34.20%	$2e^{-65}$	*Schizosaccharomyces pombe*	Q9P6J0.1
g11567.t2		1053	33.04%	$3e^{-66}$	*Schizosaccharomyces pombe*	Q9US44.1
g8046.t1	g8046	484	60.51%	0	*Postia placenta*	F1SY73.1
g8198.t1	g8198	345	62.82%	$6e^{-155}$	*Postia placenta*	F1SY73.1
g8196.t1	g8196	524	55.65%	0	*Postia placenta*	F1SY73.1
g8134.t1	g8134	448	36.71%	$9e^{-106}$	*Postia placenta*	F1SY68.1
g612.t1	g612	520	39.23%	$4e^{-108}$	*Postia placenta*	F1SYH1.1
g650.t1	g650	262	41.92%	$6e^{-67}$	*Postia placenta*	F1SY68.1
g637.t1	g637	513	41.39%	$2e^{-123}$	*Postia placenta*	F1SYH1.1
g638.t1	g638	513	40.78%	$3e^{-123}$	*Postia placenta*	F1SYH1.1
g651.t1	g651	639	36.74%	$1e^{-100}$	*Postia placenta*	F1SYH1.1
g651.t2		275	36.36%	$4e^{-38}$	*Psilocybe cubensis*	P0DPA7.1
g655.t1	g655	116	47.69%	$6e^{-16}$	*Postia placenta*	F1SYH1.1
g657.t1	g657	649	39.62%	$2e^{-102}$	*Armillaria gallica*	A0A2H3CNY6.1
g658.t1	g658	525	37.74%	$4e^{-104}$	*Postia placenta*	F1SYH1.1
g633.t1	g633	512	42.04%	$1e^{-118}$	*Postia placenta*	F1SYH1.1

g649.t1	g649	512	41.97%	$9e^{-118}$	*Postia placenta*	F1SYH1.1
g652.t1	g652	512	40.08%	$3e^{-117}$	*Postia placenta*	F1SYH1.1
g639.t1	g639	512	42.67%	$7e^{-123}$	*Postia placenta*	F1SYH1.1
g656.t1	g656	162	47.33%	$2e^{-35}$	*Armillaria gallica*	A0A2H3C NY6.1
g635.t1	g635	499	39.61%	$8e^{-113}$	*Postia placenta*	F1SYH1.1
g630.t1	g630	509	40.13%	$1e^{-119}$	*Postia placenta*	F1SYH1.1
g632.t1	g632	513	40.13%	$6e^{-119}$	*Postia placenta*	F1SYH1.1
g653.t1	g653	384	36.29%	$2e^{-85}$	*Postia placenta*	F1SY68.1
g653.t2		369	36.34%	$2e^{-77}$	*Postia placenta*	F1SYH1.1
g654.t1	g654	113	43.55%	$9e^{-13}$	*Postia placenta*	F1SYH1.1
g631.t1	g631	513	40.72%	$1e^{-118}$	*Postia placenta*	F1SYH1.1
g626.t1	g626	205	43.28%	$6e^{-45}$	*Postia placenta*	F1SYH1.1
g627.t1	g627	512	39.87%	$3e^{-112}$	*Postia placenta*	F1SYH1.1
g628.t1	g628	513	40.64%	$2e^{-115}$	*Postia placenta*	F1SYH1.1
g8152.t1	g8152	103	37.30%	$6e^{-17}$	*Postia placenta*	F1SYH1.1
g1732.t1	g1732	569	46.90%	$1e^{-151}$	*Postia placenta*	F1SYH1.1
g6524.t1	g6524	532	47.88%	$5e^{-177}$	*Aspergillus oryzae*	Q2UAZ8.1
g2460.t1	g2460	507	44.81%	$8e^{-147}$	*Aspergillus oryzae*	Q2UAZ8.1
g2458.t1	g2458	103	50.00%	$7e^{-25}$	*Postia placenta*	F1SYH1.1
g2453.t1	g2453	529	46.28%	$2e^{-161}$	*Aspergillus oryzae*	Q2UAZ8.1
g2470.t1	g2470	529	46.84%	$1e^{-164}$	*Aspergillus oryzae*	Q2UAZ8.1
g6539.t1	g6539	527	48.88%	$6e^{-180}$	*Aspergillus oryzae*	Q2UAZ8.1
g3028.t1	g3028	538	47.33%	$3e^{-144}$	*Postia placenta*	F1SYH1.1
g6570.t1	g6570	658	41.33%	$3e^{-127}$	*Postia placenta*	F1SYH1.1
g6570.t2		670	41.39%	$7e^{-135}$	*Postia placenta*	F1SYH1.1
g1406.t1	g1406	542	41.39%	$3e^{-123}$	*Postia placenta*	F1SYH1.1
g8882.t1	g8882	542	41.39%	$3e^{-123}$	*Postia placenta*	F1SYH1.1
g1408.t1	g1408	540	35.28%	$8e^{-109}$	*Coprinopsis cinerea*	A8NCK4.2
g8883.t1	g8883	540	35.28%	$8e^{-109}$	*Coprinopsis cinerea*	A8NCK4.2
g1410.t1	g1410	544	37.47%	$3e^{-114}$	*Coprinopsis cinerea*	A8NCK4.2
g8885.t1	g8885	544	37.47%	$2e^{-114}$	*Coprinopsis cinerea*	A8NCK4.2
g1409.t1	g1409	557	38.63%	$2e^{-116}$	*Postia placenta*	F1SYH1.1
g8884.t1	g8884	566	38.63%	$3e^{-116}$	*Postia placenta*	F1SYH1.1
g1513.t1	g1513	552	38.85%	$9e^{-119}$	*Postia placenta*	F1SYH1.1
g8204.t1	g8204	560	35.91%	$3e^{-100}$	*Postia placenta*	F1SY68.1
g8381.t1	g8381	507	36.57%	$1e^{-108}$	*Postia placenta*	F1SY68.1
g8408.t1	g8408	510	39.13%	$3e^{-110}$	*Postia placenta*	F1SYF1.1
g4083.t1	g4083	541	40.37%	$2e^{-125}$	*Postia placenta*	F1SYF1.1
g4087.t1	g4087	544	40.20%	$2e^{-125}$	*Postia placenta*	F1SYF1.1
g4093.t1	g4093	548	36.92%	$6e^{-114}$	*Postia placenta*	F1SYF1.1
g3247.t1	g3247	499	35.20%	$2e^{-96}$	*Postia placenta*	F1SY68.1
g3257.t1	g3257	159	42.14%	$1e^{-36}$	*Postia placenta*	F1SYF1.1
g3258.t1	g3258	330	34.28%	$6e^{-59}$	*Coprinopsis cinerea*	A8NCK4.2

g8131.t1	g8131	805	31.56%	$1e^{-81}$	*Armillaria gallica*	A0A2H3CZX2.1
g11201.t1	g11201	697	51.74%	0	*Postia placenta*	F1SY66.1
g797.t1	g797	529	36.35%	$5e^{-109}$	*Postia placenta*	F1SY62.1
g9058.t1	g9058	544	32.52%	$8e^{-73}$	*Trichoderma virens*	G9N4A8.1
g6495.t1	g6495	492	24.44%	$7e^{-26}$	*Oryctolagus cuniculus*	O02766.3
g4660.t1	g4660	99	36.78%	$1e^{-09}$	*Bos taurus*	Q29626.1
g9363.t1	g9363	518	43.43%	$2e^{-150}$	*Schizosaccharomyces pombe*	O13820.3
g10430.t1	g10430	761	32.65%	$3e^{-120}$	*Aspergillus fumigatus*	B0Y6R2.1
g10431.t1	g10431	1101	35.61%	0	*Aspergillus fumigatus*	B0Y6R2.1
g3576.t1	g3576	505	43.16%	$2e^{-128}$	*Postia placenta*	F1SYD1.1
g3577.t1	g3577	520	48.30%	$7e^{-160}$	*Postia placenta*	F1SYD1.1
g3578.t1	g3578	520	46.06%	$8e^{-158}$	*Postia placenta*	F1SYD1.1
g3579.t1	g3579	448	48.21%	$9e^{-152}$	*Postia placenta*	F1SYD1.1
g3579.t2		520	48.09%	$2e^{-159}$	*Postia placenta*	F1SYD1.1

A

>ITS of *I. hispidus*_NPCB001

TTGGGCAACTTTCCCTCCCCGTTTTTTGAATGCTTAAGTTCAGCGGGTAGTCCTACCTGATTTGAGGTCAAAGGTGTCAGAGT
TAAGGCCACTAAGGGTTCCTTGTCCTATTGCATTACAAAAAAGTAACGCGGACGATTAGAAGCAGACCCGTTAGGCAAGCG
CTCCGGTGAAGCATAGCAATACTATTACACCATAAAACGCGAACCAAAGTCCAGCTAATGCATTTAAGAGGAGCCGACAAAC
CAAAGGGCAATACAACAAAGCGTCGCCCGGGCCAGCAATAACCTCCAAGTCCAAATCGCAAGTCAAGCCTACTTCAACCAA
GAAGCAAACATAATAAAGACAAGCGATTTGAGATTAACACGACACTCAAACAGGCATGCCCCTCGGAATACCAAGGGGCGC
AAGGTGCGTTCAAAGATTCGATGATTCACTGAATTCTGCAATTCACATTACTTATCGCATTTCGCTGCGTTCTTCATCGATGCG
AGAGCCTAGAGATCCGTTGTTGAAAGTTGTACAAATTATCGCCCACAAGGAGCATTACATTCACATAACAATAAAAGGGTTT
GTAATACAAACTAGTCGAAGCGTTCGCCTTCACCCTCTCTTCTCTTAACGTTTTCCCGCTTTCAAACGTTCGAAAGGCCTCAA
AAGACCCTCCTACTAGTATTGCTCGCAGTACTAGGTCGCAAGTCGAAAGTGTTACTCACTACTAGCTTCAACCGGTTCAAAG
GTGCACAGGGGTTTGAAATGGATTTGAGCACGAAAGCCGTGCACATGTGCGTTTCCGCACCAGCACAGGCCTCCGGCTTCA
AAACTCGATAATGATCCTTCGAGTTCCCCCTCTCCAGAGAGAAGGGTTTTT

B

select all 100 sequences selected GenBank Graphics Distance tree of results MSA Viewer

Description	Scientific Name	Max Score	Total Score	Query Cover	E value	Per. Ident	Acc. Len	Accession
Inonotus hispidus clone SH2.107 small subunit ribosomal RNA gene, partial sequence; internal transcribed spacer...	Inonotus hispidus	1495	1495	96%	0.0	98.81%	848	MN699466.1
Inonotus hispidus clone SH3.103 small subunit ribosomal RNA gene, partial sequence; internal transcribed spacer...	Inonotus hispidus	1478	1478	96%	0.0	98.46%	850	MN699467.1
Inonotus hispidus clone SH1.105 small subunit ribosomal RNA gene, partial sequence; internal transcribed spacer...	Inonotus hispidus	1472	1472	96%	0.0	98.34%	848	MN699464.1
Inonotus hispidus clone SH3.104 small subunit ribosomal RNA gene, partial sequence; internal transcribed spacer...	Inonotus hispidus	1471	1471	94%	0.0	98.79%	845	MN699468.1
Inonotus hispidus clone SH1.108 small subunit ribosomal RNA gene, partial sequence; internal transcribed spacer...	Inonotus hispidus	1458	1458	93%	0.0	98.90%	850	MN699465.1
Inonotus hispidus isolate IH5D small subunit ribosomal RNA gene, partial sequence; internal transcribed spacer 1,...	Inonotus hispidus	1452	1452	94%	0.0	98.54%	845	MW074175.1
Inonotus hispidus strain BWB01 internal transcribed spacer 1, partial sequence; 5.8S ribosomal RNA gene and int...	Inonotus hispidus	1450	1450	92%	0.0	99.13%	822	MK934344.1
Inonotus hispidus strain MIL2LKP1 internal transcribed spacer 1, partial sequence; 5.8S ribosomal RNA gene and ...	Inonotus hispidus	1439	1439	92%	0.0	98.88%	806	KX881610.1

C

Inonotus hispidus clone SH2.107 small subunit ribosomal RNA gene, partial sequence; internal transcribed spacer 1, 5.8S ribosomal RNA gene, and internal transcribed spacer 2, complete sequence; and large subunit ribosomal RNA gene, partial sequence

Sequence ID: MN699466.1 Length: 848 Number of Matches: 1

Range 1: 7 to 846 GenBank Graphics ▼ Next Match ▲ Previous Match

Score	Expect	Identities	Gaps	Strand
1495 bits(809)	0.0	831/841(99%)	4/841(0%)	Plus/Minus

```
Query  14   CCTCCCCGTTTTTTGA-ATGCTTAAGTTCAGCGGGTAGTCCTACCTGATTTGAGGTCAAA  72
Sbjct  846  CCTCCGC-TTTATTGATATGCTTAAGTTCAGCGGGTAGTCCTACCTGATTTGAGGTCAAA  788
Query  73   GGTGTCAGAGTTAAGGCCACTAAGGGTTCCTTGTCCTATTGCATTACAAAAAAGTAACGC  132
Sbjct  787  GGTGTCAGAGTTAAGGCCACTAAGGGTTCCTTGTCCTATTGCATTACAAAAAAGTAACGC  728
Query  133  GGACGATTAGAAGCAGACCCGTTAGGCAAGCGCTCCGGTGAAGCATAGCAATACTATTAC  192
Sbjct  727  GGACGATTAGAAGCAGACCCGTTAGGCAAGCGCTCCGGTGAAGCATAACCATACTATTAC  668
Query  193  ACCATAAAACGCGAACCAAAGTCCAGCTAATGCATTTAAGAGGAGCCGACAAACCAAAGG  252
Sbjct  667  ACCATAAAACGCGAACCAAAGTCCAGCTAATGCATTTAAGAGGAGCCGACAAACCAAAGG  608
Query  253  GCAATACAACAAAGCGTCGCCCGGGCCAGCAATAACCTCCAAGTCCAAATCGCAAGTCAA  312
Sbjct  607  GCAATACAACAAAGCGTCGCCCGGGCCAGCAATAACCTCCAAGTCCAAATCGCAAGTCAA  548
Query  313  GCCTACTTCAACCAAGAAGCAAACATAATAAAGACAAGCGATTTGAGATTAACACGACAC  372
Sbjct  547  GCCTACTTCAACCAAGAAGCAAACATAATAAAGACAAGCGATTTGAGATTAACACGACAC  488
Query  373  TCAAACAGGCATGCCCCTCGGAATACCAAGGGGCGCAAGGTGCGTTCAAAGATTCGATGA  432
Sbjct  487  TCAAACAGGCATGCCCCTCGGAATACCAAGGGGCGCAAGGTGCGTTCAAAGATTCGATGA  428
Query  433  TTCACTGAATTCTGCAATTCACATTACTTATCGCATTTCGCTGCGTTCTTCATCGATGCG  492
Sbjct  427  TTCACTGAATTCTGCAATTCACATTACTTATCGCATTTCGCTGCGTTCTTCATCGATGCG  368
Query  493  AGAGCCTAGAGATCCGTTGTTGAAAGTTGTACAAATTATCGCCCACAAGGAGCATTACAT  552
Sbjct  367  AGAGCCTAGAGATCCGTTGTTGAAAGTTGTACAAATTATCGCCCACAAGGAGCATTACAT  308
Query  553  TCACATAACAATAAAAGGGTTTGTAATACAAACTAGTCGAAGCGTTCGCCTTCACCCTCT  612
Sbjct  307  TCACATAACAATAAAAGGGTTTGTAATACAAACTAGTCGAAGCGTTCGCCTTCACCCTCT  248
Query  613  CTTCTCTTAACGTTTTCCCGCTTTCAAACGTTCGAAAGGCCTCAAAAGACCCTCCTACTA  672
Sbjct  247  CTTCTCTTAACGTTTTCCCGCTTTCAAACGTTCGAAAGGCCTCAAAAGACCCTCCTACTA  188
```

Figure S1. ITS alignment of the strain NPCB_001.

A. ITS sequence of the strain NPCB_001. B. Comparison results of ITS of the strain NPCB_001 based on NCBI blastP. C. The ITS of strain NWAFU-1 was aligned to *Inonotus hispidus* clone SH2.107 (MN699466.1).

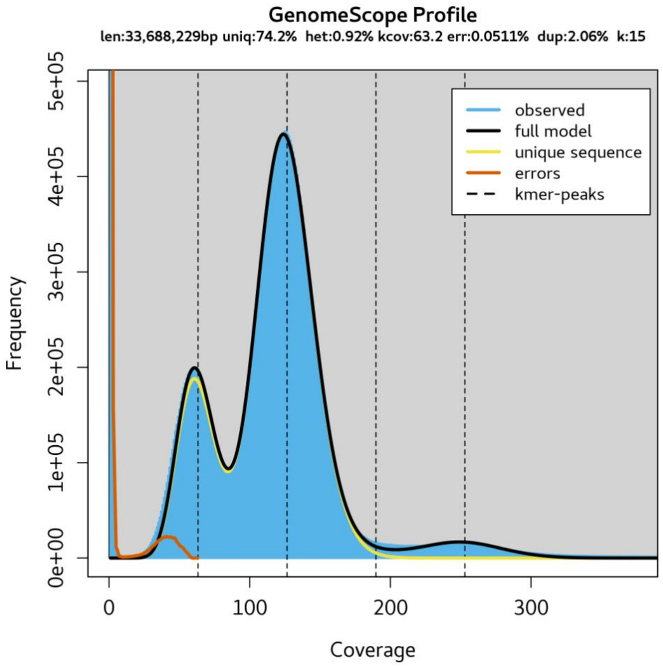

Figure S2. *K*-mer Depth and *K*-mer Species Frequency Distribution Plot.
The blue line represents the actual *K*-mer curve, the black line is the k-mer curve estimated by the model, the yellow line is the *K*-mer curve corresponding to the unique data, the red line represents the error curve due to sequencing errors, and the dashed line represents speculation *K*-mer peak. Using the reads obtained by sequencing, *K*-mer-based analysis was used to estimate the genome size and heterozygosity. A *K*-mer refers to a sequence of K bp in length. Iteratively select a sequence of length *K* bases from a continuous sequence. If the length of the sequence is L and the length of the *K*-mer is K, then L-*K*+1 *K*-mers can be obtained. We take K-mers for the reads obtained by sequencing, and then count the frequency of each *K*-mer. According to the Lander waterman algorithm, the genome size (G) satisfies the following formula:

$$C_{base} = C_{k-mer} \times \frac{L}{L-K+1}$$

$$G = \frac{n_{k-mer}}{C_{k-mer}} = \frac{n_{base}}{C_{base}}$$

C_{base} and $C_{k\text{-mer}}$ are the expected depth of coverage and *K*-mer, and nbase and n*k*-mer are the total number of bases and the total number of *K*-mers in the sequence. In the case of a certain amount of data, the depth frequency of *K*-mer is subject to Poisson distribution, so the peak of the *K*-mer depth frequency distribution is the corresponding depth, which is used as an estimate of the expected depth of *K*-mer.

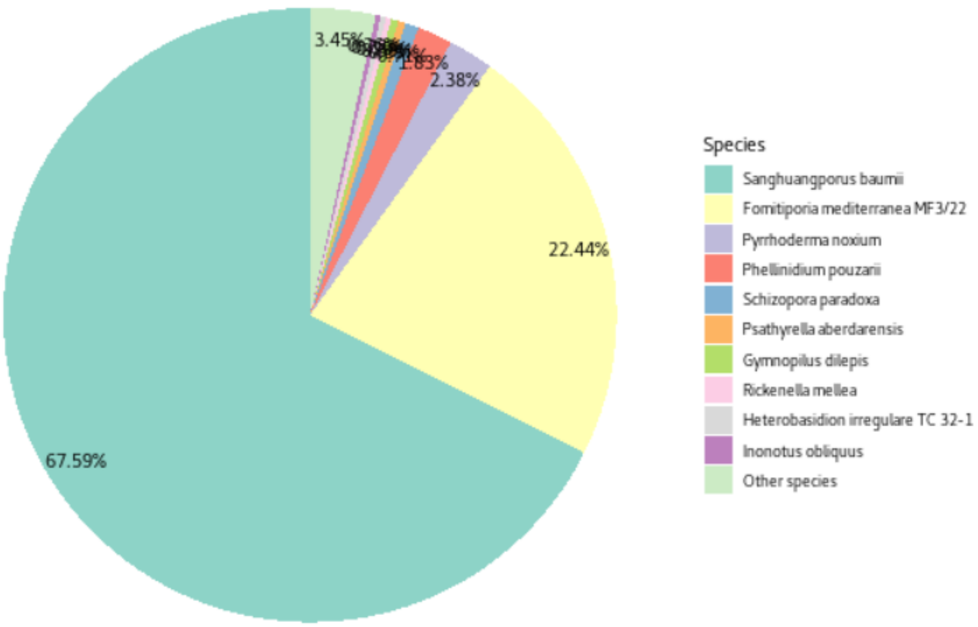

Figure S3. Species distribution map of Nr database alignment to sequences.

According to the results of the Nr library match, the top 10 species were counted and the rest were classified as other species, and the distribution of these species was mapped according to their proportion.

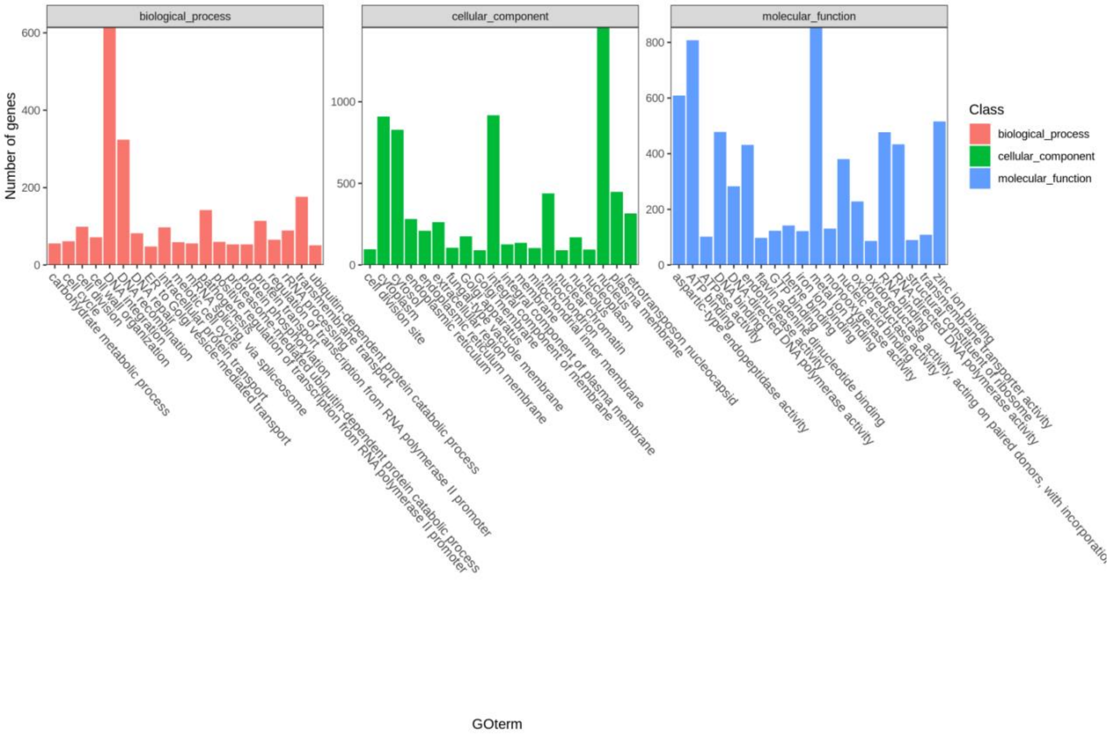

Figure S4. Statistical map of functional annotation classification based on Gene Ontology database.

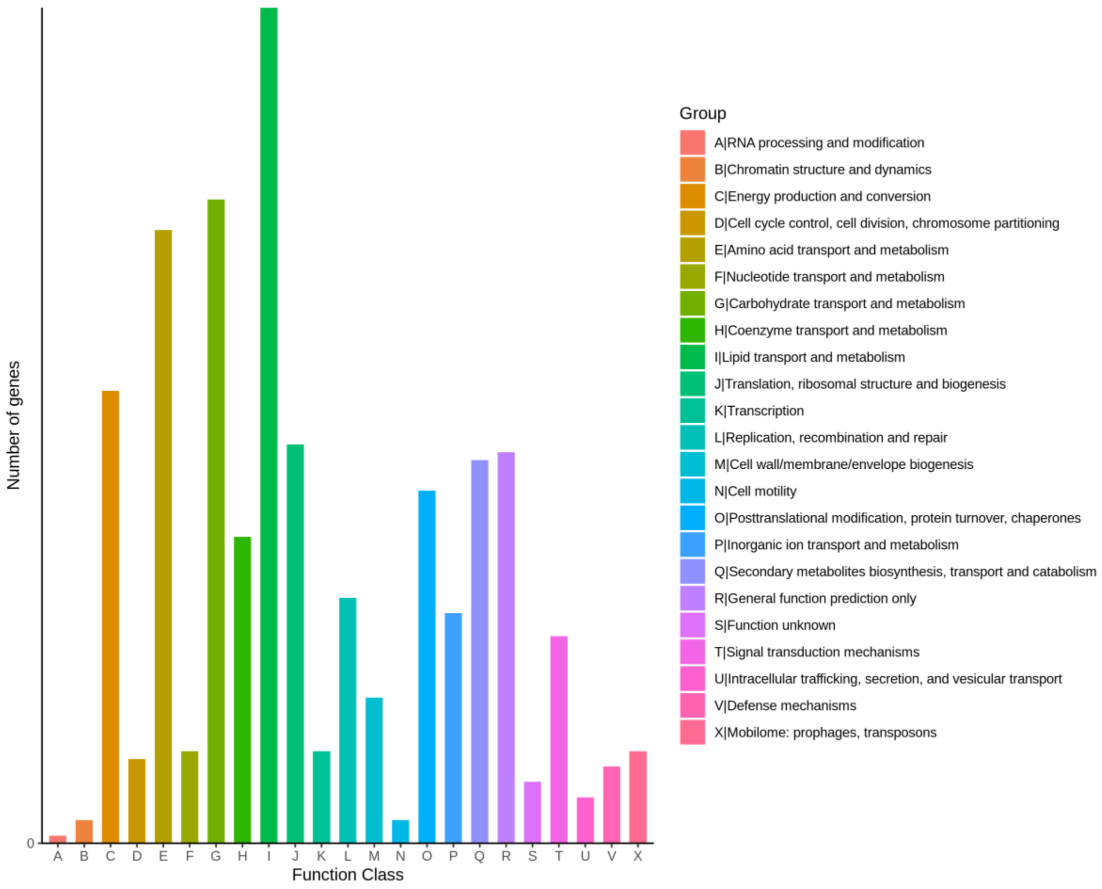

Figure S5. Statistical Chart of COG Functional Annotated Classification.

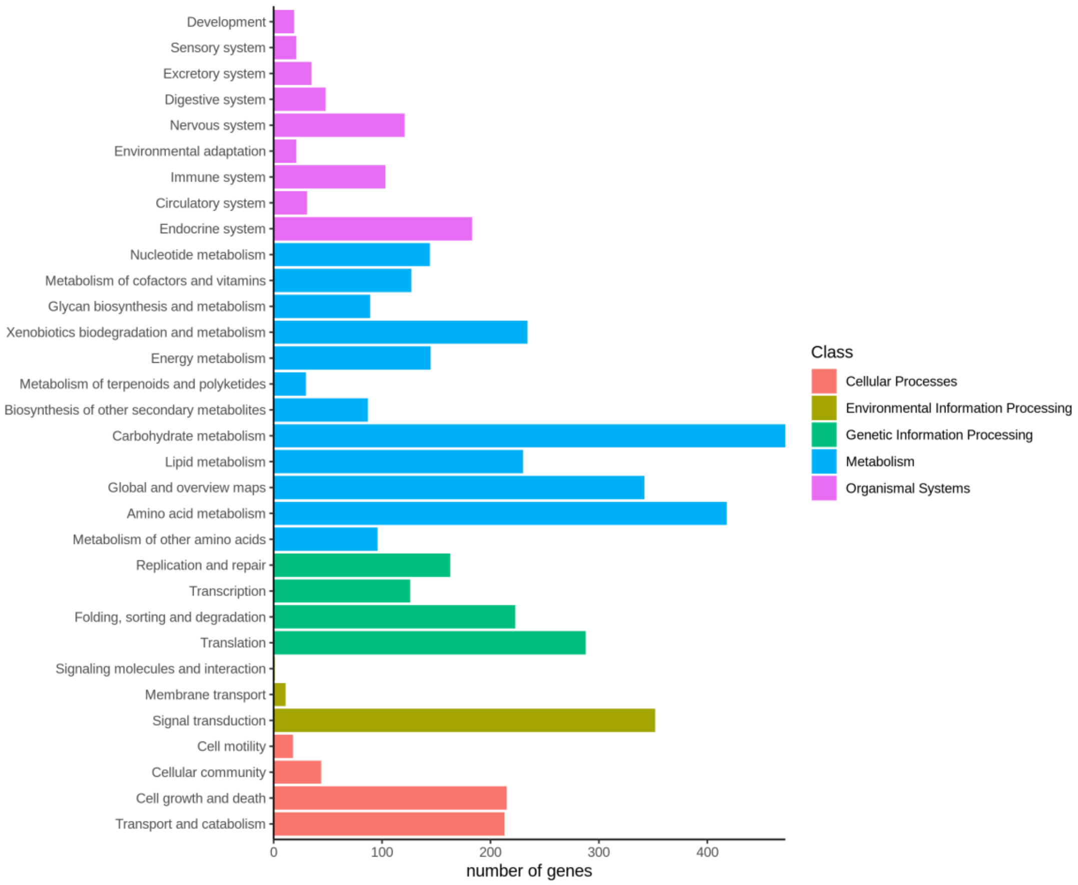

Figure S6. KEGG Pathway Functional Classification Chart.

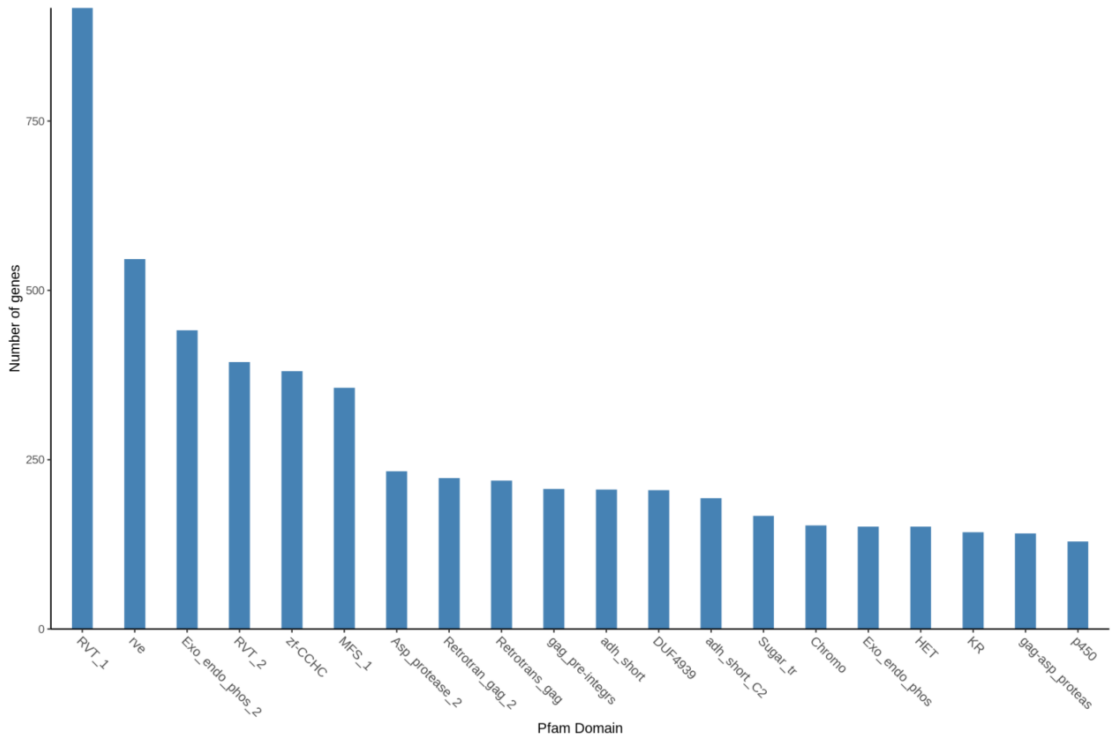

Figure S7. Domain annotation based on the Pfam database.

Statistical summaries of genes annotated for each domain are made and the top 20 most annotated domains are plotted.

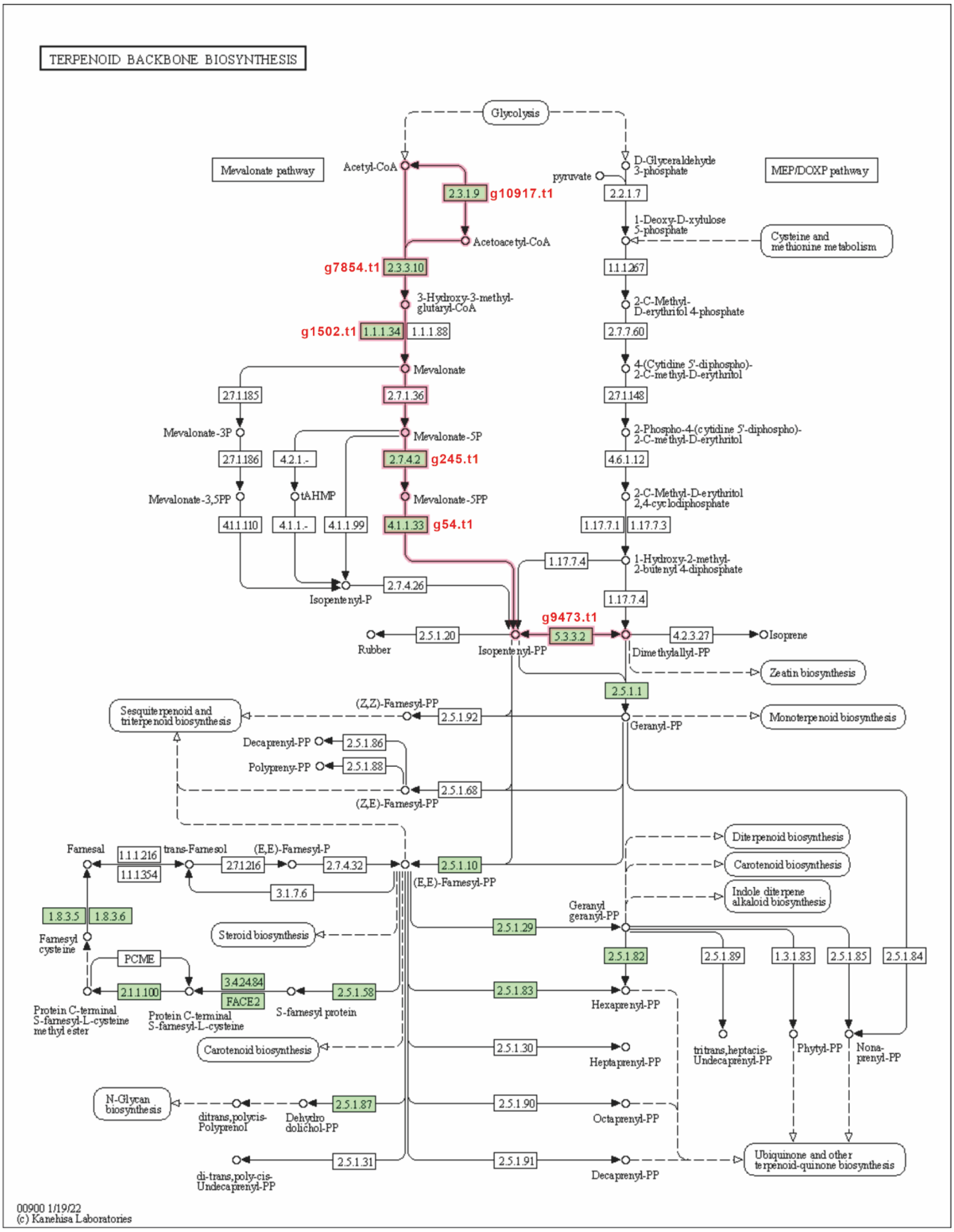

Figure S8. Annotation of the terpenoid backbone biosynthesis of NPCB_001 using KAAS.

The genes with green background represent NPCB_001-derived genes annotated into the pathway (00900), and proteins with red font represent specific NPCB_001-derived proteins involved in MVP. KAAS annotation is done by KEGG Automatic Annotation Server Ver. 2.1.

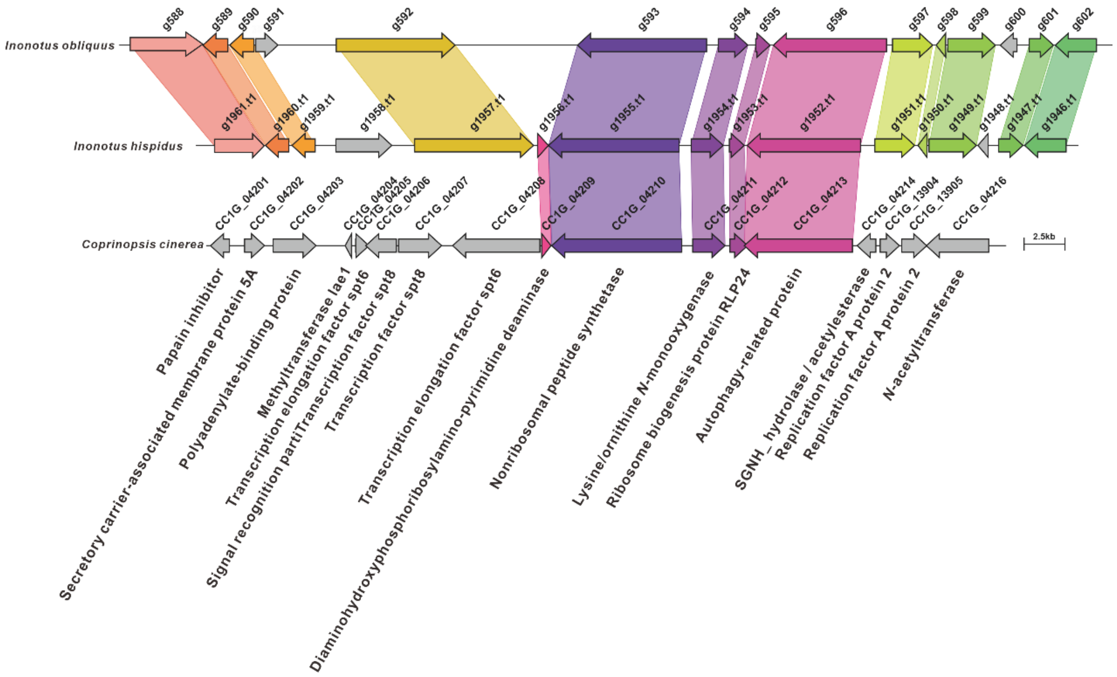

Figure S9. Functional analysis of NRPS and comparative analysis of the BGCs involved.

The four core genes (CCG_04210-CCG_04213) on the BGC for siderophore of *Coprinopsis cinerea*[1] are highly conserved with their counterparts in *Inonotus* species. Similarity comparison of these gene clusters was done by clinker[2].

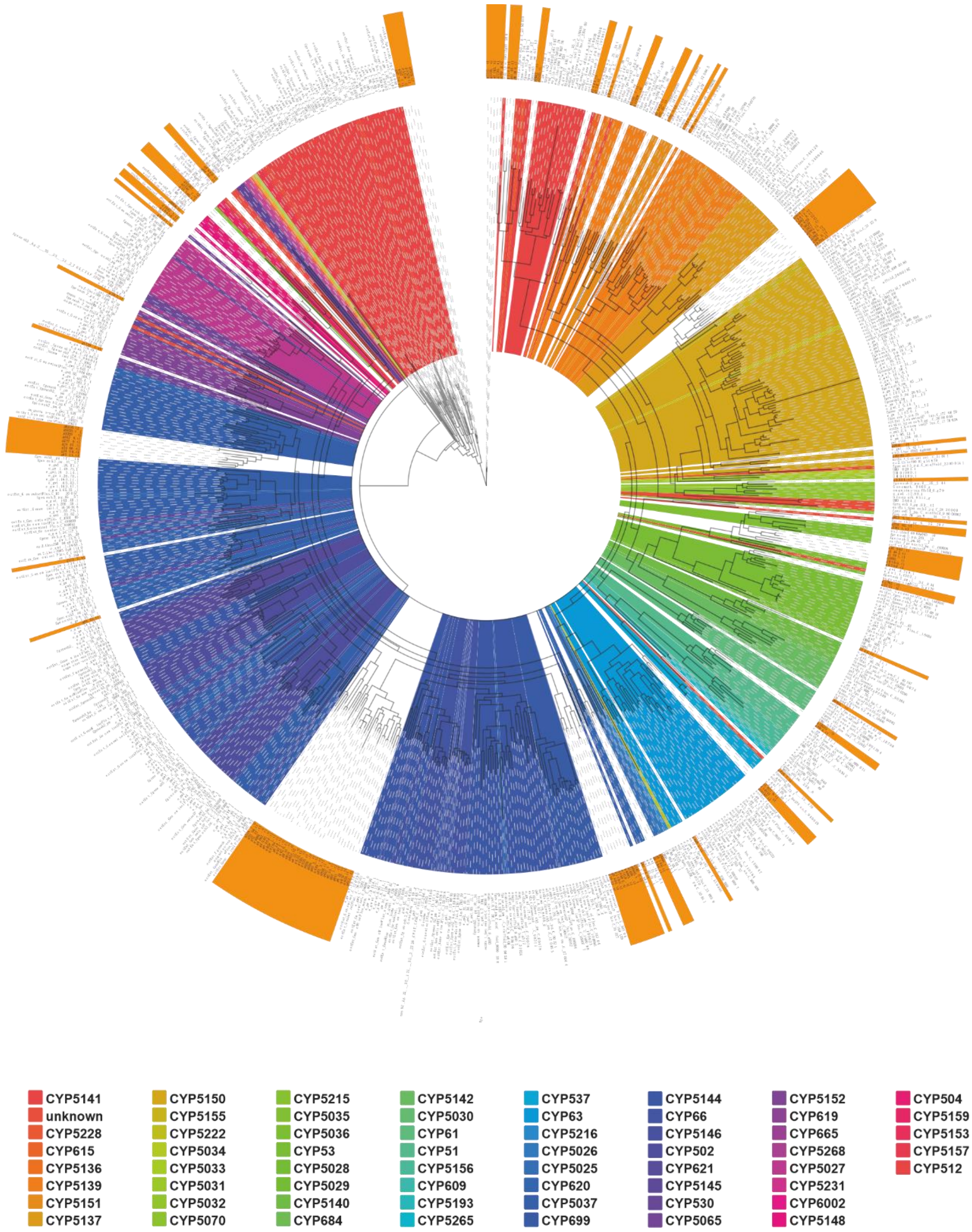

Figure S10. P450s Cluster analysis of the strain *I. hispidus* NPCB_001 and other Basidiomycetes.

The sequences of the branch with color background and the corresponding sequence name without background are from Fungal Cytochrome P450 Database (http://passport.riceblast.snu.ac.kr/?t=P450). The background color of the branch corresponds to the category to which it belongs. The sequences with dark green background and corresponding branch without background are P450 sequences from *I. hispidus* NPCB_001. Multiple sequence pairs are implemented with mafft[3] v7.505 with parameters --maxiterate 1000 --localpair. The evolutionary tree was constructed by IQtree[4] v2.2.3 with the parameters -m MFP -bb 1000 -alrt 1000 -abayes -nt AUTO.

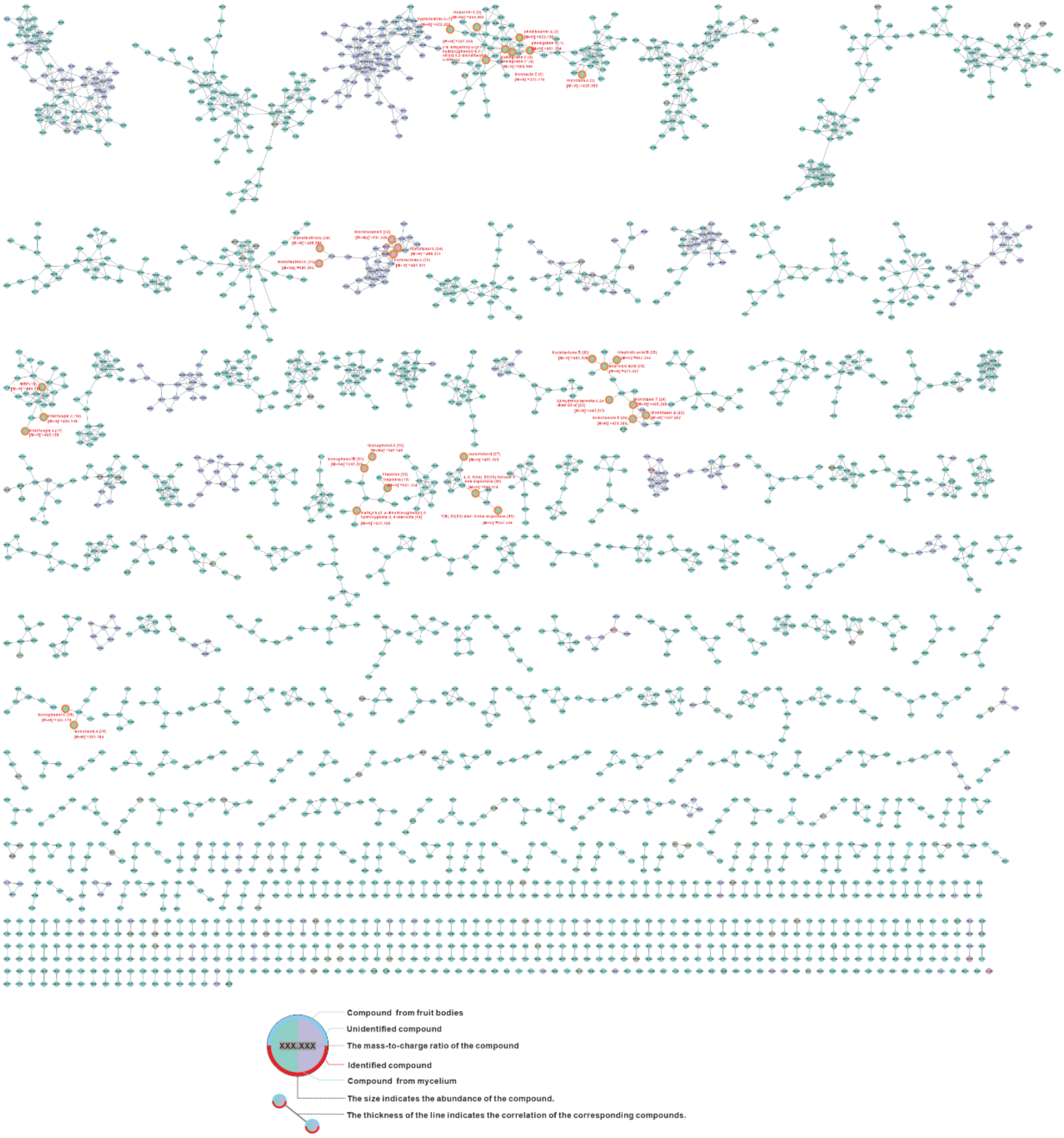

Figure S11. Molecular network analysis of metabolites from the mycelium and fruiting bodies of the strain *I. hispidus* NPCB_001.

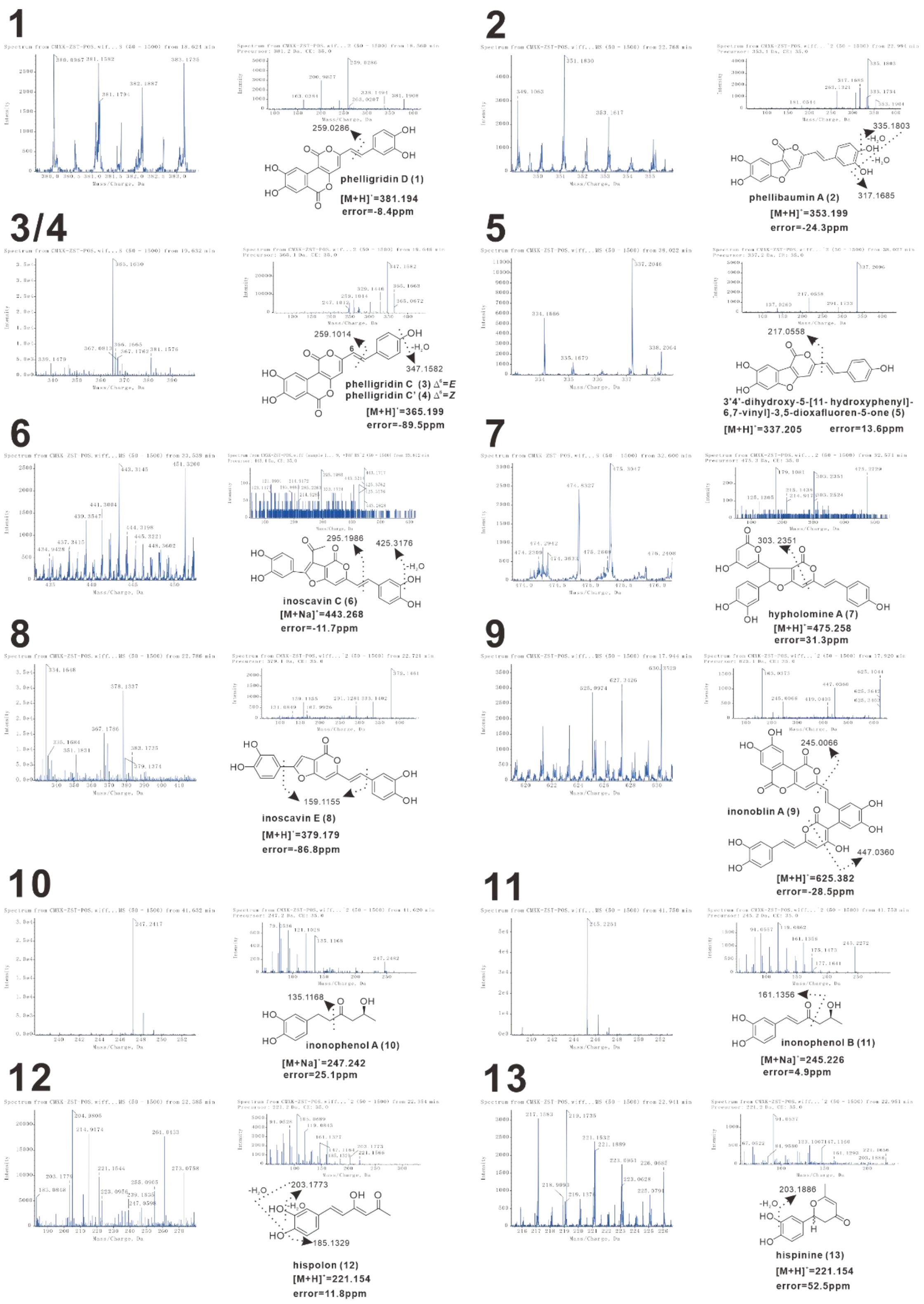
1
259.0286
phelligridin D (1)
[M+H]+=381.194
error=-8.4ppm
2
335.1803
317.1685
phellibaumin A (2)
[M+H]+=353.199
error=-24.3ppm
3/4
259.1014
347.1582
phelligridin C (3) Δ6=E
phelligridin C' (4) Δ6=Z
[M+H]+=365.199
error=-89.5ppm
5
217.0558
3'4'-dihydroxy-5-[11- hydroxyphenyl]-6,7-vinyl]-3,5-dioxafluoren-5-one (5)
[M+H]+=337.205
error=13.6ppm
6
295.1986
425.3176
inoscavin C (6)
[M+Na]+=443.268
error=-11.7ppm
7
303.2351
hypholomine A (7)
[M+H]+=475.258
error=31.3ppm
8
159.1155
inoscavin E (8)
[M+H]+=379.179
error=-86.8ppm
9
245.0066
447.0360
inonoblin A (9)
[M+H]+=625.382
error=-28.5ppm
10
135.1168
inonophenol A (10)
[M+Na]+=247.242
error=25.1ppm
11
161.1356
inonophenol B (11)
[M+Na]+=245.226
error=4.9ppm
12
203.1773
185.1329
hispolon (12)
[M+H]+=221.154
error=11.8ppm
13
203.1886
hispinine (13)
[M+H]+=221.154
error=52.5ppm

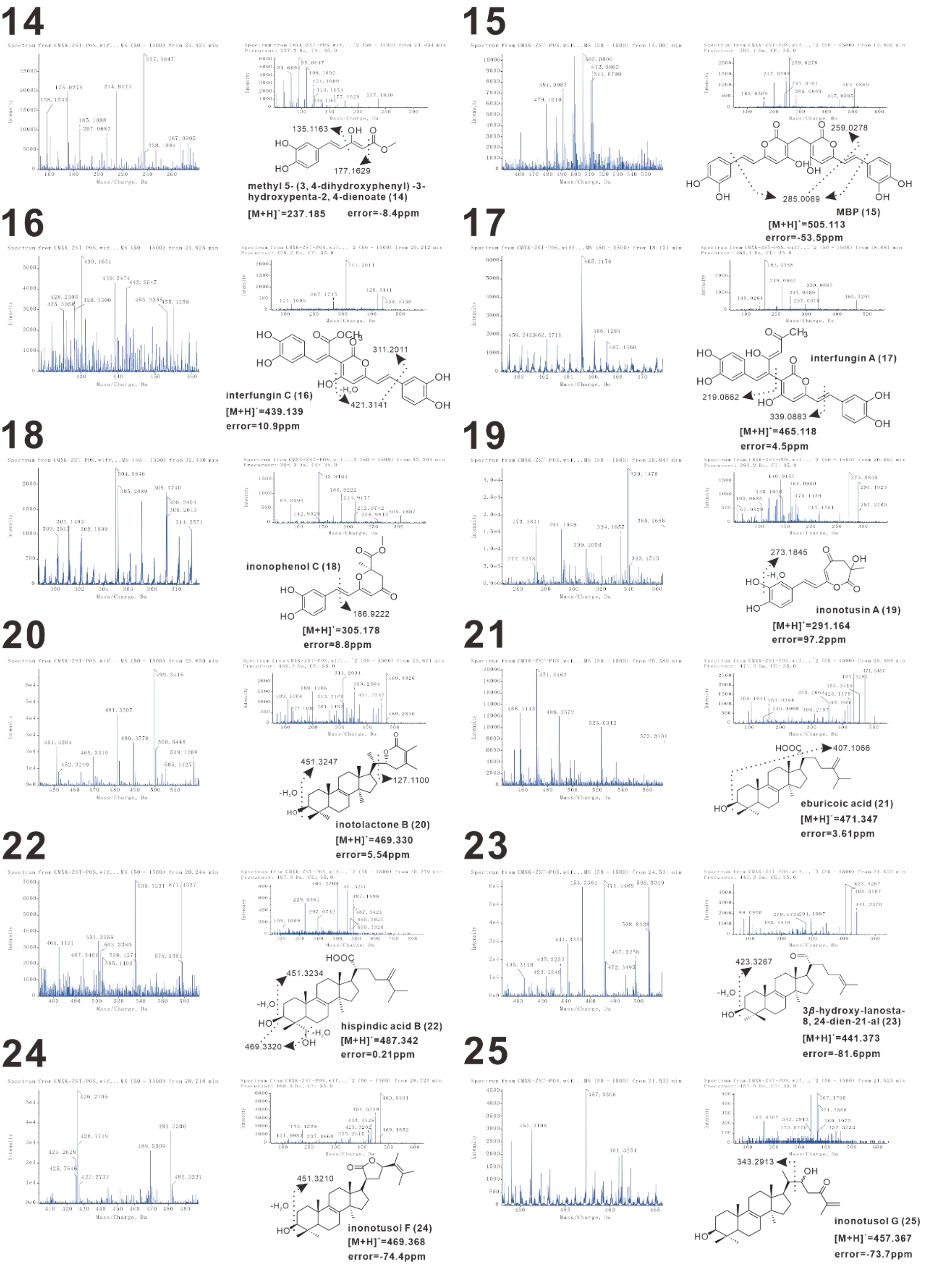
14
135.1163
177.1629
methyl 5- (3, 4-dihydroxyphenyl) -3-hydroxypenta-2, 4-dienoate (14)
[M+H]+=237.185 error=-8.4ppm
15
259.0278
285.0069
MBP (15)
[M+H]+=505.113
error=-53.5ppm
16
311.2011
421.3141
interfungin C (16)
[M+H]+=439.139
error=10.9ppm
17
interfungin A (17)
219.0662
339.0883
[M+H]+=465.118
error=4.5ppm
18
inonophenol C (18)
186.9222
[M+H]+=305.178
error=8.8ppm
19
273.1845
inonotusin A (19)
[M+H]+=291.164
error=97.2ppm
20
451.3247
127.1100
inotolactone B (20)
[M+H]+=469.330
error=5.54ppm
21
407.1066
eburicoic acid (21)
[M+H]+=471.347
error=3.61ppm
22
451.3234
469.3320
hispindic acid B (22)
[M+H]+=487.342
error=0.21ppm
23
423.3267
3β-hydroxy-lanosta-8, 24-dien-21-al (23)
[M+H]+=441.373
error=-81.6ppm
24
451.3210
inonotusol F (24)
[M+H]+=469.368
error=-74.4ppm
25
343.2913
inonotusol G (25)
[M+H]+=457.367
error=-73.7ppm

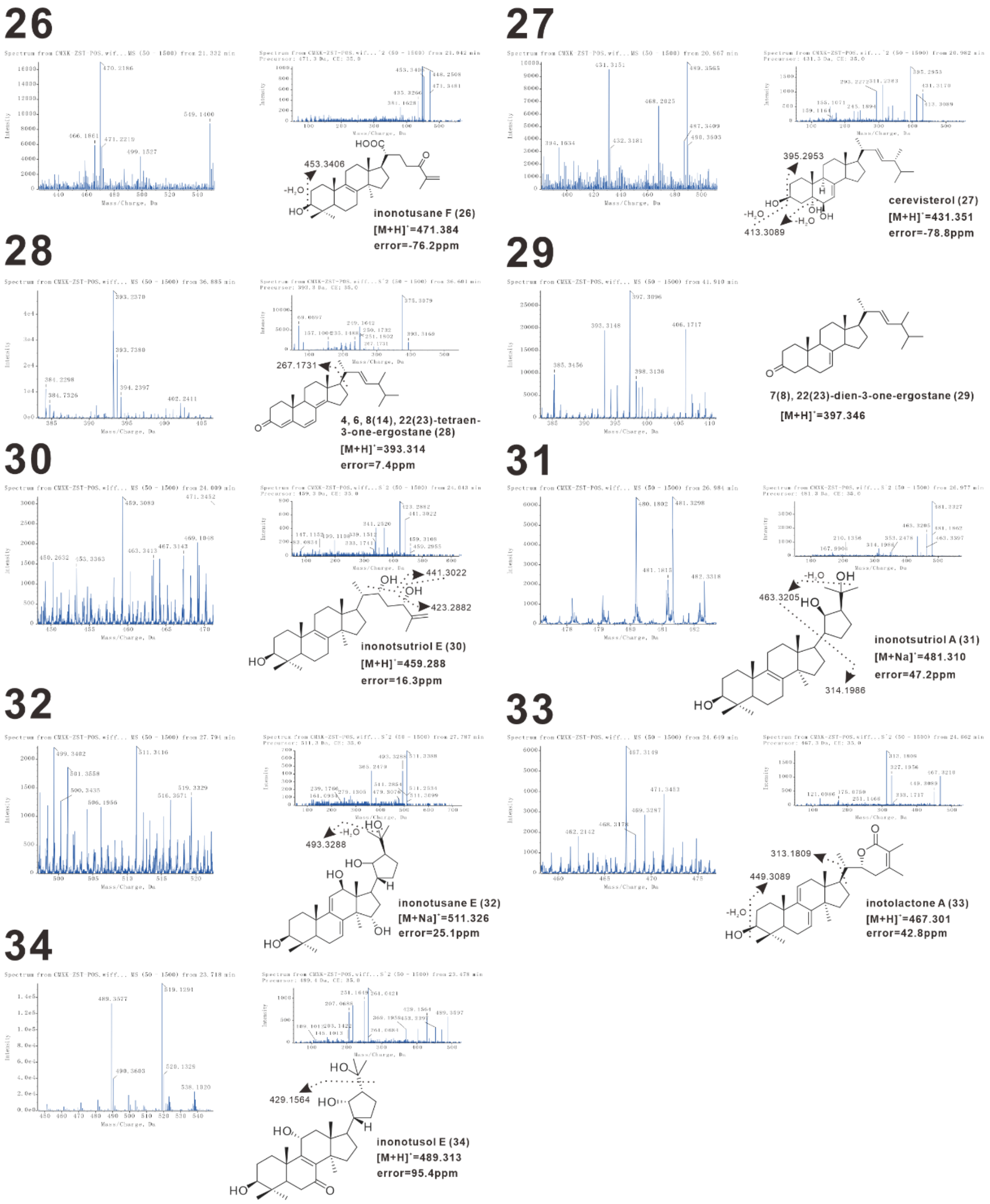

Figure S12. The LC-ESI-HRMS and LC-ESI-HRMS/MS spectrums of isolates from the strain *I. hispidus* NPCB_001.

Mass spectral data were acquired in positive ion mode. The spectrum numbers correspond to the compound numbers in the manuscript.

Reference

1. Tsunematsu, Y., et al., *Genomic Mushroom Hunting Decrypts Coprinoferrin, A Siderophore Secondary Metabolite Vital to Fungal Cell Development.* Organic Letters, 2019. **21**(18): p. 7582-7586.
2. Gilchrist, C.L.M. and Y.-H. Chooi, *clinker & clustermap.js: automatic generation of gene cluster comparison figures.* Bioinformatics, 2021. **37**(16): p. 2473-2475.
3. Nakamura, T., et al., *Parallelization of MAFFT for large-scale multiple sequence alignments.* Bioinformatics, 2018. **34**(14): p. 2490-2492.
4. Minh, B.Q., et al., *IQ-TREE 2: New Models and Efficient Methods for Phylogenetic Inference in the Genomic Era.* Molecular Biology and Evolution, 2020. **37**(5): p. 1530-1534.
